# Supplementary material for: Safeguarding China’s long-term sustainability against systemic disruptors
Source: Nat Commun. 2024 Jun 24;15:5338. doi: 10.1038/s41467-024-49725-9 (PMC11196269; doi:10.1038/s41467-024-49725-9)
Supplement: Supplementary file 1 — Supplementary Information [file 41467_2024_49725_MOESM1_ESM.pdf]

Supplementary Information for

Safeguarding China's long-term sustainability against systemic disruptors

Ke Li [1], Lei Gao [2], Zhaoxia Guo [1], Yucheng Dong [1,*], Enayat A. Moallemi [3], Gang Kou [4,5], Meiqian Chen [1], Wenhao Lin [1], Qi Liu [1], Michael Obersteiner [6,7], Matteo Pedercini [8], Brett A. Bryan [9]

[1] Business School, Sichuan University, Chengdu 610065, P.R. China.

[2] Commonwealth Scientific and Industrial Research Organisation (CSIRO), Waite Campus, Adelaide, South Australia 5064, Australia.

[3] Commonwealth Scientific and Industrial Research Organisation (CSIRO), Black Mountain, ACT, Australia

[4] Xiangjiang Laboratory, Changsha 410205, China.

[5] School of Business Administration, Faculty of Business Administration, Southwestern University of Finance and Economics, Chengdu 610074, China.

[6] International Institute for Applied Systems Analysis, Laxenburg, 2361, Austria.

[7] The Environmental Change Institute, University of Oxford, UK.

[8] Millennium Institute, Washington, DC, USA.

[9] Centre for Integrative Ecology, School of Life and Environmental Sciences, Deakin University, Melbourne, Australia.

* Corresponding author. E-mail: ycdong@scu.edu.cn

This file includes:

Supplementary Methods

Supplementary Figures 1 to 20

Supplementary Tables 1 to 7

Supplementary References

Supplementary Methods

1. A brief summary of disruptors

Five main disruptors were identified, including pandemic disease, ageing and shrinking population, deglobalization, climate change and biodiversity loss. Each of these disruptors presents unique consequences. To better understand the disruptors, we provide a brief summary covering the likelihood of disruptors occurring in the coming years, key drivers behind the disruptors, and their potential consequences. The parameters associated with the identified disruptors and their uncertainties are presented in Supplementary Table 1.

Pandemic disease. This disruptor refers to emerging or re-emerging infectious disease epidemics and pandemics, resulting in serious health crises and economic recession. The COVID-19 pandemic led to the loss of life[1–3], pressure on the health system, a severe economic shock[4], and employment and well-being were adversely affected[5–7]. Although the COVID-19 pandemic has now passed, the risk of resurgence remains a significant concern[8]. Several factors can contribute to a resurgence including the emergence of variants of the virus[9] and decreasing population immunity[10] over time. Climate change, combined with rapid urbanization and changing land-use patterns may also increase the likelihood of new pandemic diseases emerging[11].

Ageing and shrinking population. Population ageing is a summary term for shifts in the age distribution (i.e., age structure) of a population toward older ages[12], which arises from longer life expectancy and declining fertility rates. Between 1950 and 2020, global life expectancy rose from 46 to 73 years and the fertility rate fell from 5.0 to 2.4[13], resulting in a threefold increase in the number of persons aged 60 years and over[14]. For China, the number of people aged 60 years and over reached 264 million in 2020, accounting for over 18.5% of China's total population[15]. At the same time, population size is challenged by declining fertility rate. Many countries and regions are projected to experience significant population shrinking by 2050[16]. China's growth of population has slowed due to the implementation of the one-child policy in 1980, which dropped from 2.5% in 1970 to 0.7% in 2000. And data show that China's population has already peaked and is beginning to shrink[17].

This huge demographic shift poses severe threats to public health and economic development[16,18]. As the population ages and shrinks, there is a reduction in the proportion of working-age people and an increased workforce for older adults' care, resulting in sharp falls in employment[19,20]. The increased proportion of old workers (e.g., 55-64 years) can reduce labour productivity due to the decrease of physical attributes with age[21]. Moreover, there are public health challenges associated with a rapidly ageing population, including the escalating costs in terms of care and medicine[22], and the increased need for social services[23] and pensions[24], which require more expenditure for health care, leading to the redirection of government

expenditure from other sectors or increased fiscal deficits.

Deglobalization. The term “deglobalization” arose as a counterpoint to globalization. It refers to the process of “diminishing interdependence and integration between certain units around the world”[25], characterized by reduced international trade, decreased foreign capital flows, and increased protectionist policies[26]. In recent years, there has been increasing evidence of this trend, as countries prioritize their national interests over global collaboration[27]. For example, foreign direct investment, an indicator to measure economic globalization, experienced a sharp decline. According to the United Nations Conference on Trade and Development (UNCTAD), foreign direct investment flows fell by 42% in 2020[28], reaching the lowest level since the 1990s. Current disruptors, such as the COVID-19 pandemic and the Russian invasion of Ukraine, has further contributed to deglobalization by exacerbating existing trends and introducing new challenges. Economic downturns and energy crises during the period not only undermined support for globalization, but have also prompted national governments to re-evaluate external dependencies and prioritized self-sufficiency production to foster the resilience against external shocks[29]. Increasing uncertainties further discouraged foreign investment and weakened regional economic integration[30].

China has long been heavily dependent on foreign investment and international trade. Deglobalization would pose several challenges to economic development, such as decreased export demand[31], reduced access to foreign investment[31], and slowed productivity growth. Furthermore, deglobalization would have implications for China's politics and society. Slowing economic growth caused by deglobalization could affect social stability, leading to increased unemployment and widening income inequality[32].

Climate change. Several direct consequences of climate change are highlighted in the literature, including higher energy consumption due to persistent reliance on fossil fuels and inefficient energy use[33], exacerbating water scarcity due to changes in the distribution, frequency and intensity of rainfall events[34], rising global temperatures[33] and increased frequency and intensity of extreme weather events[35]. Indirect environmental consequences such as rising sea levels[36], ocean acidification[37], and loss of biodiversity[38] are likely to follow. These consequences have broad implications for human societies, economies and ecosystems.

Although there have been advances in addressing climate change such as an increasing share of renewable energy and improved climate adaptation measures, global efforts currently risk falling short of achieving the Paris Agreement’s objectives[39]. A variety of factors hinder climate action. The shift toward renewable energy and low-carbon technologies requires significant investment which can be challenging for developing economies. Governments face multiple competing interests and may prioritize short-term economic gains over long-term sustainability.

Biodiversity loss. Biodiversity is the variety of life, including variation among genes, species, and functional traits[40]. There is ample evidence suggesting that biodiversity is

being lost at an alarming rate and biodiversity loss has become a pressing global concern[40,41], with far-reaching consequences for ecosystems, human societies, and economies. The decline in biodiversity affects the functioning of ecosystems and their ability to provide essential goods and services, such as food production, water purification, and climate regulation, which are crucial for human well-being and economic prosperity[42].

Various factors contribute to biodiversity loss, including habitat destruction and fragmentation, overexploitation, environment pollution and climate change[43–45]. In China, deforestation and habitat loss have played a substantial role in biodiversity loss. Land conversion for agriculture and infrastructure development resulted in the destruction of habitats for many plant and animal species[46]. Rapid urbanization and industrial development have also led to increased pollution, resource exploitation, and habitat fragmentation, further contributing to biodiversity loss[47]. However, as China moves toward more sustainable development practices, it has taken actions to mitigate the challenges associated with biodiversity loss[48]. The future of biodiversity is receiving policy attention in China via significant resource allocation toward land-use and biodiversity conversation.

2. Construction of pandemic module within iSDG-China model

To analyse the impact of the COVID-19 pandemic or future pandemic diseases on the progress towards the SDGs, we further advanced the iSDG-China model by developing a pandemic module and extending 6 existing modules (i.e., population, employment, balance of payments, government, vehicles, and emissions & waste) based on the interlinkages among these modules and pandemic module (Supplementary Fig. 19). In the following subsections, the description of pandemic module and how pandemic module interacts with other modules are provided.

Pandemic module. Pandemic module represents how the mitigation policies, vaccinations and the evolution of virus variants are combined to determine the transmission of the COVID-19 pandemic or other pandemic diseases in the future. The infectious disease modelling is a tool that has been used to capture the mechanisms by which infectious diseases spread, to predict the future course and to evaluate strategies to control diseases. A classic model is the Susceptible-Infected-Recovered (SIR) model[49], which divides the population into three groups: susceptible, infectious and recovered, and uses differential equations to describe the quantitative relationships among the three groups. There are many models derived from the SIR model, including the Susceptible-Exposed-Infectious-Recovered (SEIR) model where there is a latent period of the disease during which the individuals exposed is not infectious. These models are widely used in past pandemics, such as SARS[50] and SARS-CoV-2[51–54]. Informed by these existing studies[51,53,55], the basic SEIR model is used to capture the transmission of the pandemic in China while incorporates vaccine-induced population immunity and the containment measures for mobility, including reducing the contact rate between individuals, shutting down national borders and keeping infected

individual under quarantine. Supplementary Fig. 20 illustrates the structure of pandemic module.

The inputs of pandemic module include the transmission of virus, the infection-fatality rate of virus, and the level of containment measures. The key outputs include the number of COVID-19 infections, deaths, hospitalizations and the number of individuals under quarantine. The key assumptions of pandemic module are as follows: (1) The population from other countries to China is considered, but the population from China to other countries is not simulated; (2) Individuals under quarantine cannot contact with susceptible individuals; (3) Individuals vaccinated or recovered from the pandemic could gain immunity, but immunity only lasts for a certain period of time and individuals would be at risk of a reinfection after this period.

Interactions between pandemic and population module. Population module simulates total population and age distribution with 101 age cohorts (age 0 to 100+) for both genders. The COVID-19 pandemic would take a severe human toll if the resurgence of massive infections occurs[48]. Due to the differences in age and sex dependency of COVID-19 mortality, our extension of population module could calculate the potential annal deaths due to COVID-19 and total population by age and gender based on sex- and age-specific infection-fatality rates of China[53].

Interactions between pandemic and employment module. Employment module represents how economic activity creates employment. In the iSDG model, the employment is determined by capital and years of schooling. The pandemic-related disruptions have an unprecedented impact on employment. In order to capture this impact, our extension of the employment module calculates working time loss by individuals who have not been infected but were placed under quarantine, as well as confirmed cases and individuals not considered to have had COVID-19. Together these form the total work time loss.

Interactions between pandemic and balance of payments module. The balance of payments module is contained in economy modules and calculates the level of exports which is determined by the country's productivity and the level of taxes on international trade in the iSDG model. The pandemic-related disruptions have affected trade flows around the world. To capture the pandemic impact on exports, our extension of the balance of payments module calculates export loss, which is driven by total work time loss, a proxy for the severity of supply chain disruption.

Interactions between pandemic and government module. Government module calculates government revenue and government expenditure. Government expenditure is categorized into education, health, biodiversity and other interventions. Our extension of government module calculates the government expenditure increase due to the pandemic, including treatment expenditure for confirmed cases, quarantine expenditure for suspected cases and vaccination expenditure.

Interactions between pandemic and vehicles module. Vehicles module represents

the number of passenger and freight vehicles, and the CO_2 emissions and PM2.5 concentrations generated by these vehicles. To prevent the spread of the pandemic, governments have adopted strict containment measures, which have resulted in restrictions on travel and border closures. In order to capture the pandemic impacts on transport sector, our extension of vehicles module calculates transport loss, which is driven by contact rate between individuals, a proxy for the mobility of the specific country.

Interactions between pandemic and emissions & waste module. Emissions & waste module calculates greenhouse gas emissions and solid waste. More waste has been generated during the pandemic. To capture this impact, our extension of the emissions & waste module calculates waste increase due to the pandemic, including treatment-related medical waste and face mask use.

3. Calculation of economic output within iSDG model

In brief, total economic output calculation is defined as the sum of the economic output of agriculture, industry and services sectors, formulated by Equation (1), using an extended Cobb-Douglas production function for each sector. A detailed description of economic output calculation (e.g., major assumptions, exogenous input variables, initialization variables and source literature) is available in the iSDG model documentation[56].

$$GDP = EO_{agr} + EO_{ind} + EO_{ser} \qquad \text{Equation (1)}$$

where GDP denotes gross domestic product; and EO_{agr}, EO_{ind}, and EO_{ser} denote the economic output of agriculture, industry and services sectors, respectively.

The agriculture production is formulated as follows.

$$EO_{agr} = EO_{crops} + EO_{livestock} + EO_{forestry} + EO_{fishery} \qquad \text{Equation (2)}$$

where EO_{crops}, $EO_{livestock}$, $EO_{forestry}$ *and* $EO_{fishery}$ denote crops production, livestock production, forestry production and fishery production, respectively. Production factors of the agriculture production include land, capital and labour, and factor productivity depends on several other drivers, including: infrastructure, education, health, governance, access to electricity, macroeconomic stability, female participation in the workforce, openness to trade, climate change, energy prices and public expenditure in agriculture sector.

The industry production is formulated as follows.

$$EO_{ind} = TFP_{ind} L_{ind}^{1-\beta} K_{ind}^{\beta} \qquad \text{Equation (3)}$$

where L_{ind} and K_{ind} are the labour and capital of the industry sector; and β denotes the elasticity coefficient of capital-output of the industry sector; and TFP_{ind}

is total factor productivity for the industry sector, which depends on several drivers including: infrastructure, education, health, governance, access to electricity, macroeconomic stability, female participation in the workforce, openness to trade, climate change, and energy prices.

The services production is formulated as follows.

$$EO_{ser} = TFP_{ser} L_{ser}^{1-\gamma} K_{ser}^{\gamma} \qquad \text{Equation (4)}$$

where L_{ser} and K_{ser} are the labour and capital of the services sector; and γ denotes the elasticity coefficient of capital-output of the services sector; and TFP_{ser} is total factor productivity for the services sector, which depends on several drivers including: infrastructure, education, health, governance, access to electricity, macroeconomic stability, female participation in the workforce, openness to trade, climate change, and energy prices.

A detailed calculation of the agriculture, industry and services production is available in the iSDG model documentation[56].

Supplementary Figures

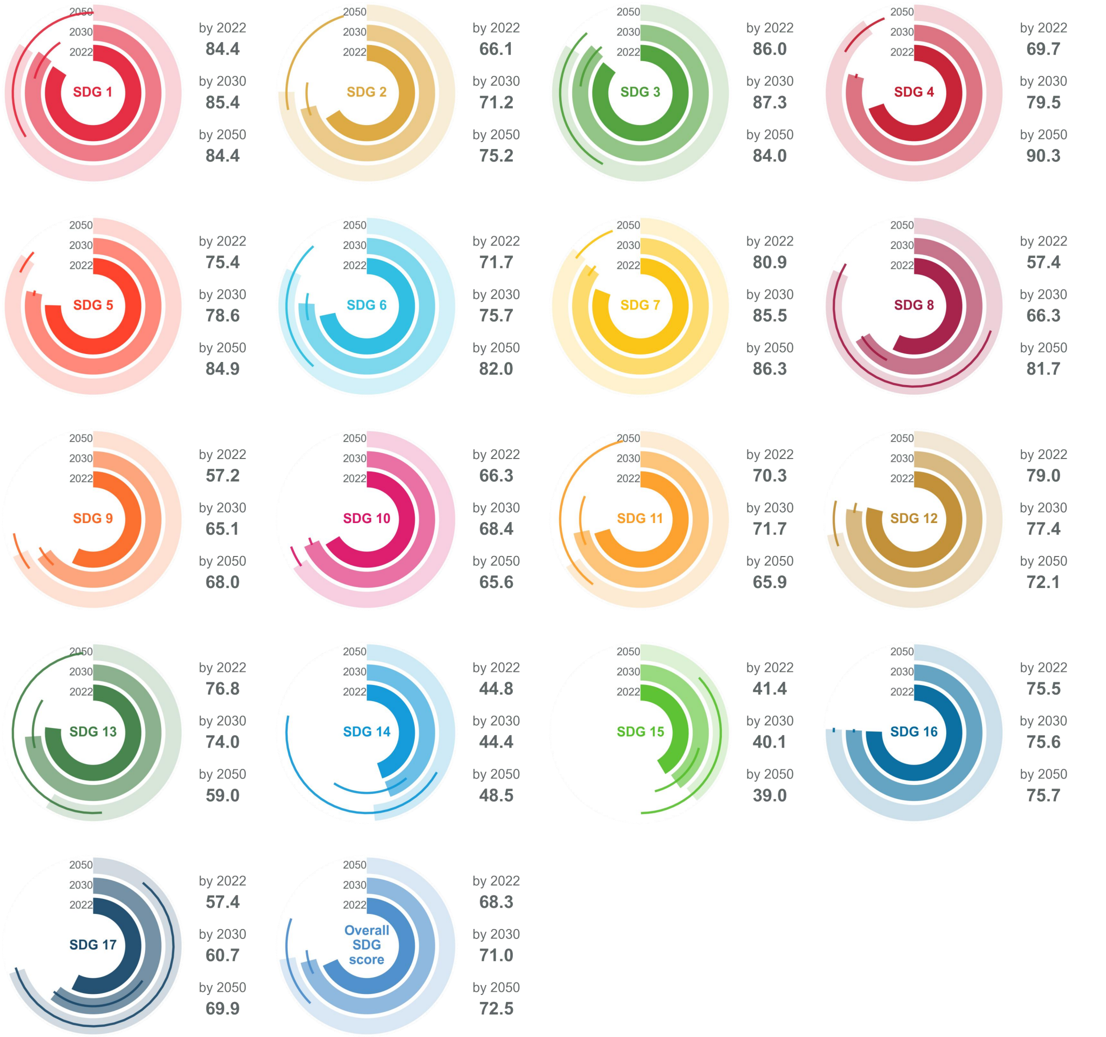

Supplementary Figure 1. Individual SDG scores and overall SDG scores in 2022, 2030 and 2050 under the Baseline scenario. The annotated figures are the individual SDG scores or overall SDG scores. In each panel, the three bars show the individual SDG scores or overall SDG scores in 2022, 2030 and 2050, respectively. And the line in each bar (i.e., error bar) shows the range of the individual SDG scores or overall SDG scores of all scenarios. This figure is reproduced, with permission, from figure 5 of Moallemi et al[57].

SDGs	Variables	2022	Baseline	Pandemic disease	Ageing and shrinking population	Deglobalization	Climate change	Biodiversity loss	Compound effects
1&3	Average access to basic health care (worst 0–1 best)	0.805	0.897	0.896	0.737	0.797	0.903	0.898	0.652
3	Life expectancy at birth	78.21	81.09	80.96	80.5	80.67	81.12	81.1	79.99
4	Total average years of schooling (years)	9.78	12.15	12.16	11.96	12.06	12.16	12.16	11.91
6	Water resources vulnerability index (worst 0–1 best)	23.91	27.23	27.18	23.05	24.81	33.56	27.25	24.52
8	Real GDP per capita (2015 thousand Yuan/person)	70.2	284.6	283.8	188.1	212.3	292.7	285.2	149.4
12	Material footprint per capita (t)	24.03	65.73	65.58	47.8	52.29	67.49	65.84	40.27
13	GHG emissions (Gt)	10.00	15.66	15.58	9.43	11.37	18.72	15.7	8.77
14	Proportion of territorial waters effectively protected (%)	3.78	6.06	6.03	3.74	4.54	6.22	4.63	2.25
15	Forest cover (%)	20.40	23.38	23.22	21.33	21.62	23.34	21.23	18.84
15	Red list index (worst 0–1 best)	0.73	0.68	0.68	0.68	0.68	0.66	0.68	0.64
17	Government surplus or deficit (% of GDP)	-0.04	-0.54	-0.54	-0.9	-0.86	-0.52	-0.47	-1.27
–	Government revenue (trillion Yuan)	23.4	241.0	239.9	145.4	179.2	247.9	241.6	114.9
–	Working-age population (billion persons aged 15–65)	1.01	0.84	0.83	0.78	0.83	0.84	0.84	0.78
–	Exports (% of GDP)	23.7	18.4	18.4	13.6	15.6	18.8	18.4	12.1

Supplementary Figure 2. The simulated results of key indicators which are significantly affected by the disruptors by 2050. See Supplementary Table 7 for more simulated results of SDG indicators.

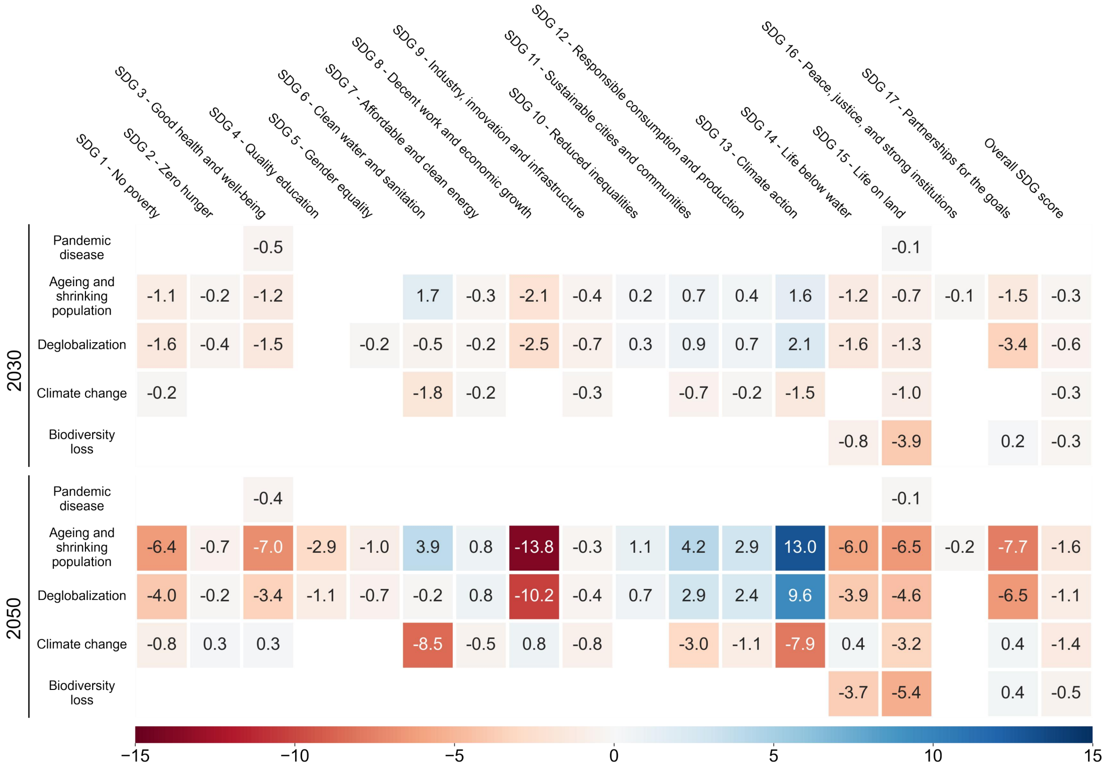

Supplementary Figure 3. The main effects of individual disruptors on the SDGs over the medium (2030) to long (2050) term. The coloured shading indicates main effects of individual disruptors on individual SDG scores and overall SDG scores. See Methods for the detailed calculation process of main effects. Cells are blank where the differences were small (i.e., between -0.1 and 0.1).

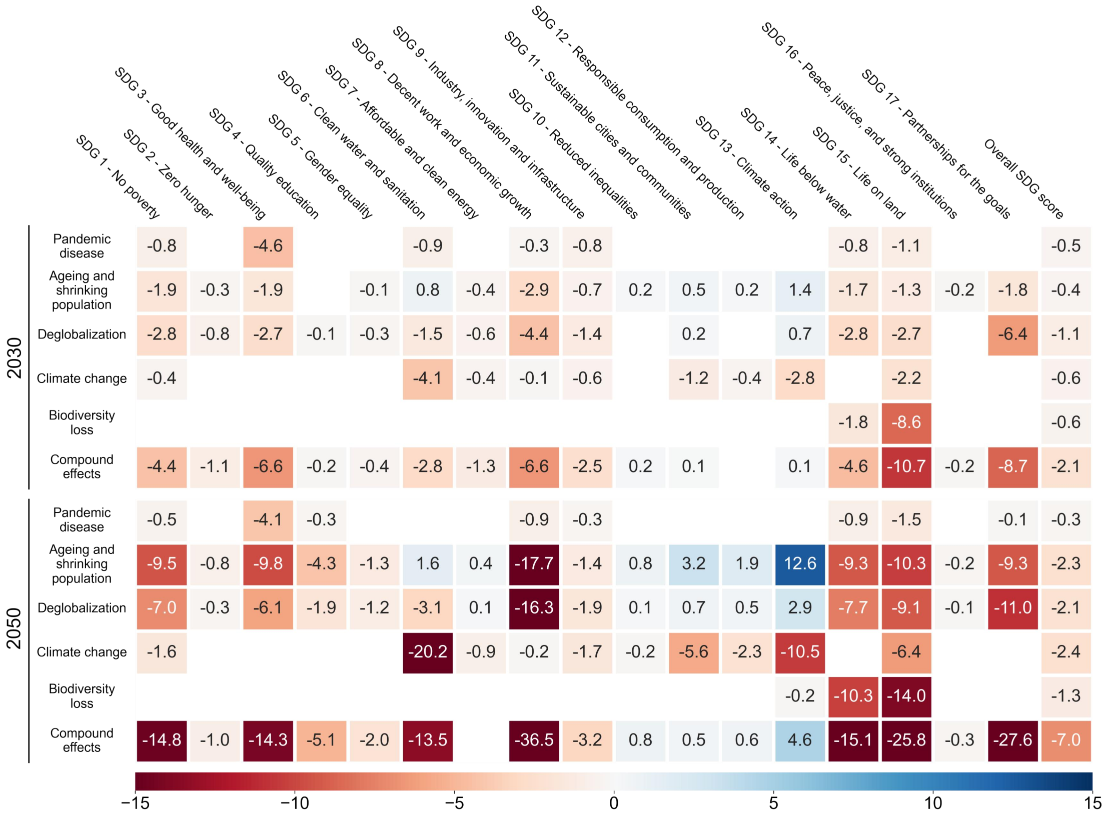

Supplementary Figure 4. The maximum impacts of individual disruptors and the compound effects on the SDGs over the medium (2030) to long (2050) term. The coloured shading indicates their performance differences as compared against the Baseline scenario in individual SDG scores and overall SDG scores. Cells are blank where the differences were small (i.e., between -0.1 and 0.1).

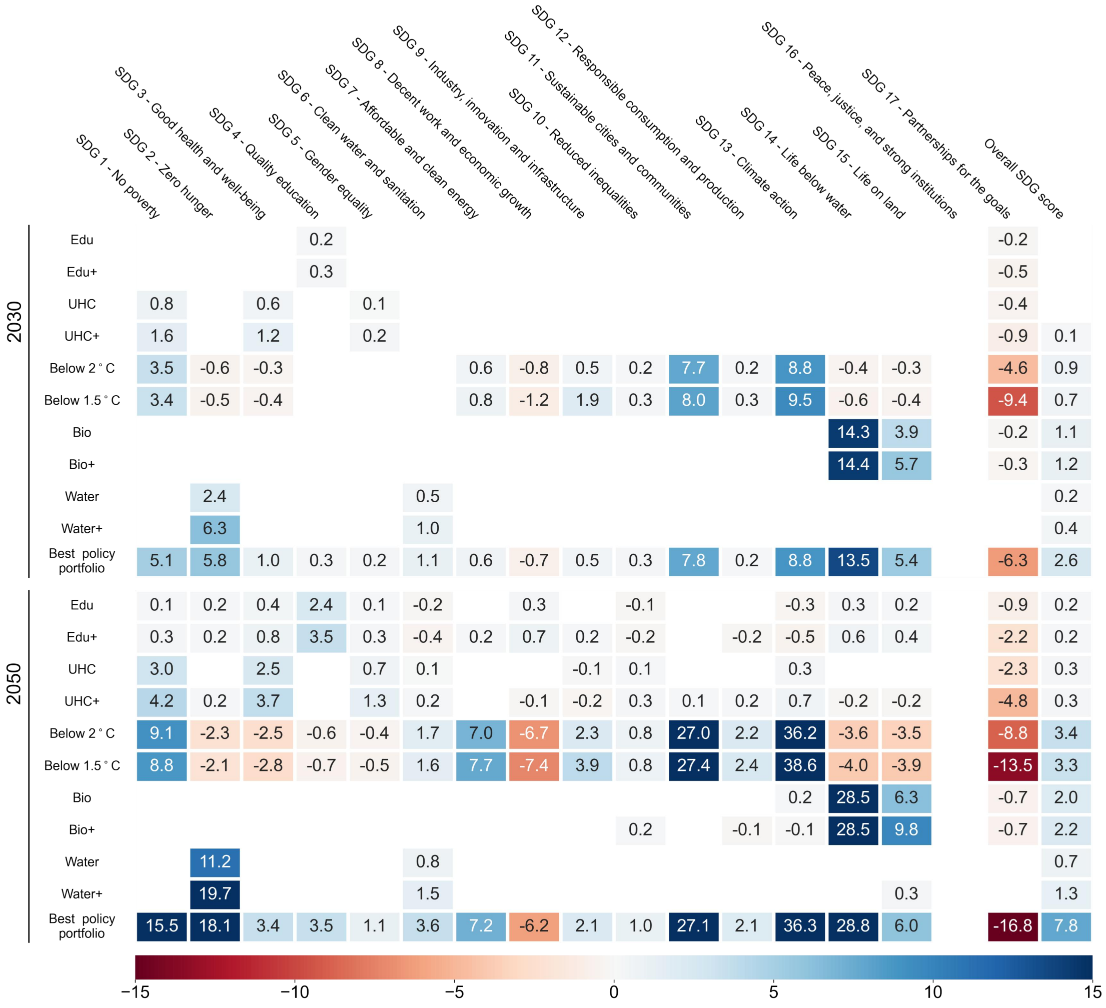

Year	Policy	SDG 1 - No poverty	SDG 2 - Zero hunger	SDG 3 - Good health and well-being	SDG 4 - Quality education	SDG 5 - Gender equality	SDG 6 - Clean water and sanitation	SDG 7 - Affordable and clean energy	SDG 8 - Decent work and economic growth	SDG 9 - Industry, innovation and infrastructure	SDG 10 - Reduced inequalities	SDG 11 - Sustainable cities and communities	SDG 12 - Responsible consumption and production	SDG 13 - Climate action	SDG 14 - Life below water	SDG 15 - Life on land	SDG 16 - Peace, justice, and strong institutions	SDG 17 - Partnerships for the goals	Overall SDG score
2030	Edu				0.2													-0.2	
	Edu+				0.3													-0.5	
	UHC	0.8		0.6		0.1												-0.4	
	UHC+	1.6		1.2		0.2												-0.9	0.1
	Below 2 ° C	3.5	-0.6	-0.3				0.6	-0.8	0.5	0.2	7.7	0.2	8.8	-0.4	-0.3		-4.6	0.9
	Below 1.5 ° C	3.4	-0.5	-0.4				0.8	-1.2	1.9	0.3	8.0	0.3	9.5	-0.6	-0.4		-9.4	0.7
	Bio														14.3	3.9		-0.2	1.1
	Bio+														14.4	5.7		-0.3	1.2
	Water		2.4				0.5												0.2
	Water+		6.3				1.0												0.4
	Best policy portfolio	5.1	5.8	1.0	0.3	0.2	1.1	0.6	-0.7	0.5	0.3	7.8	0.2	8.8	13.5	5.4		-6.3	2.6
2050	Edu	0.1	0.2	0.4	2.4	0.1	-0.2		0.3		-0.1			-0.3	0.3	0.2		-0.9	0.2
	Edu+	0.3	0.2	0.8	3.5	0.3	-0.4	0.2	0.7	0.2	-0.2		-0.2	-0.5	0.6	0.4		-2.2	0.2
	UHC	3.0		2.5		0.7	0.1			-0.1	0.1			0.3				-2.3	0.3
	UHC+	4.2	0.2	3.7		1.3	0.2		-0.1	-0.2	0.3	0.1	0.2	0.7	-0.2	-0.2		-4.8	0.3
	Below 2 ° C	9.1	-2.3	-2.5	-0.6	-0.4	1.7	7.0	-6.7	2.3	0.8	27.0	2.2	36.2	-3.6	-3.5		-8.8	3.4
	Below 1.5 ° C	8.8	-2.1	-2.8	-0.7	-0.5	1.6	7.7	-7.4	3.9	0.8	27.4	2.4	38.6	-4.0	-3.9		-13.5	3.3
	Bio													0.2	28.5	6.3		-0.7	2.0
	Bio+										0.2		-0.1	-0.1	28.5	9.8		-0.7	2.2
	Water		11.2				0.8												0.7
	Water+		19.7				1.5									0.3			1.3
	Best policy portfolio	15.5	18.1	3.4	3.5	1.1	3.6	7.2	-6.2	2.1	1.0	27.1	2.1	36.3	28.8	6.0		-16.8	7.8

Supplementary Figure 5. Synergies and trade-offs of single-policy portfolios and the best policy portfolio (Edu+, UHC+, Below 2°C, Bio+ and Water+) compared to the baseline policy portfolio under the states of the world with pandemic disease by 2030 and 2050. The coloured shading indicates under the states of the world with pandemic disease, their performance differences as compared the baseline policy portfolio in individual SDG scores and overall SDG scores. Cells are blank where the differences were small (i.e., between -0.1 and 0.1).

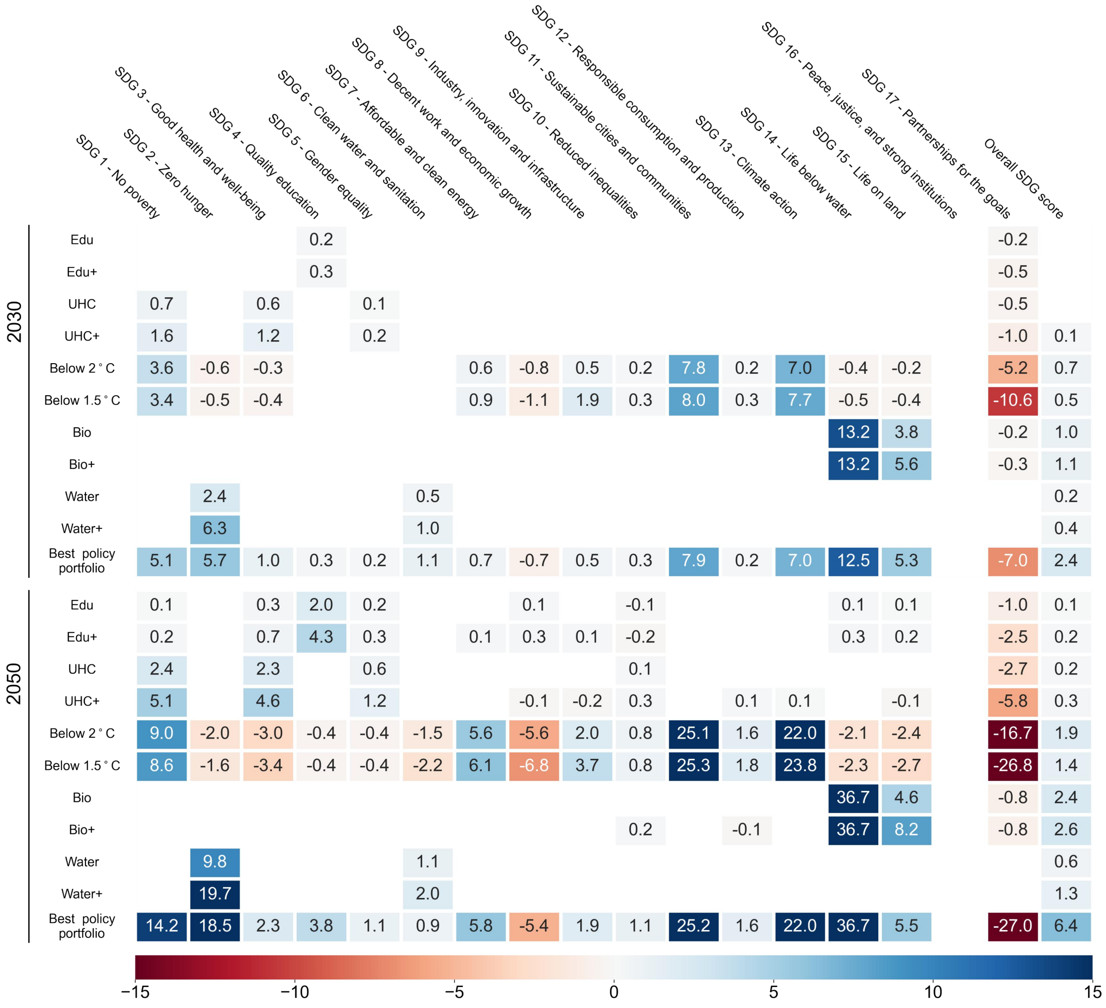

Supplementary Figure 6. Synergies and trade-offs of single-policy portfolios and the best policy portfolio (Edu+, UHC+, Below 2°C, Bio+ and Water+) compared to the baseline policy portfolio under the states of the world with ageing and shrinking population by 2030 and 2050. The coloured shading indicates under the states of the world with ageing and shrinking population, their performance differences as compared the baseline policy portfolio in individual SDG scores and overall SDG scores. Cells are blank where the differences were small (i.e., between -0.1 and 0.1).

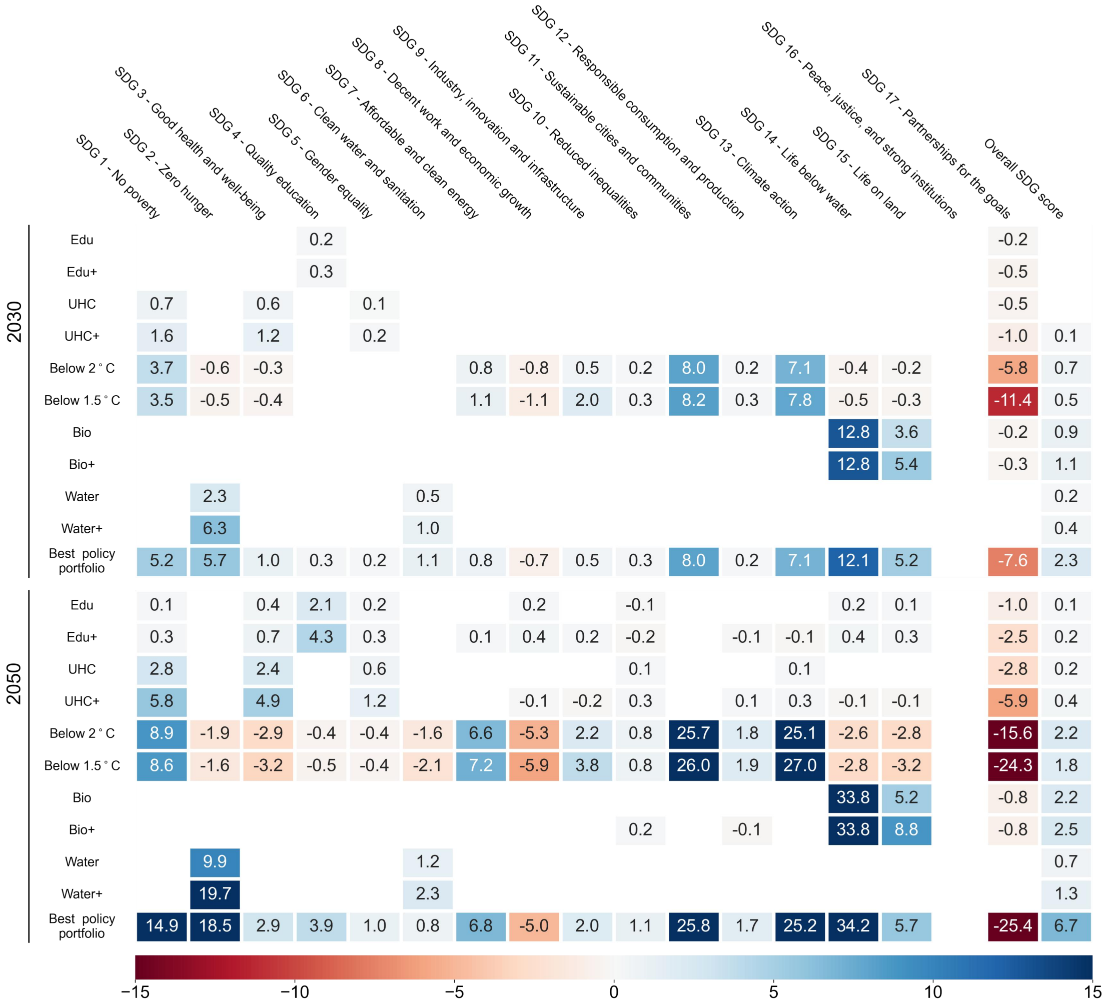

Supplementary Figure 7. Synergies and trade-offs of single-policy portfolios and the best policy portfolio (Edu+, UHC+, Below 2°C, Bio+ and Water+) compared to the baseline policy portfolio under the states of the world with deglobalization by 2030 and 2050. The coloured shading indicates under the states of the world with deglobalization, their performance differences as compared the baseline policy portfolio in individual SDG scores and overall SDG scores. Cells are blank where the differences were small (i.e., between -0.1 and 0.1).

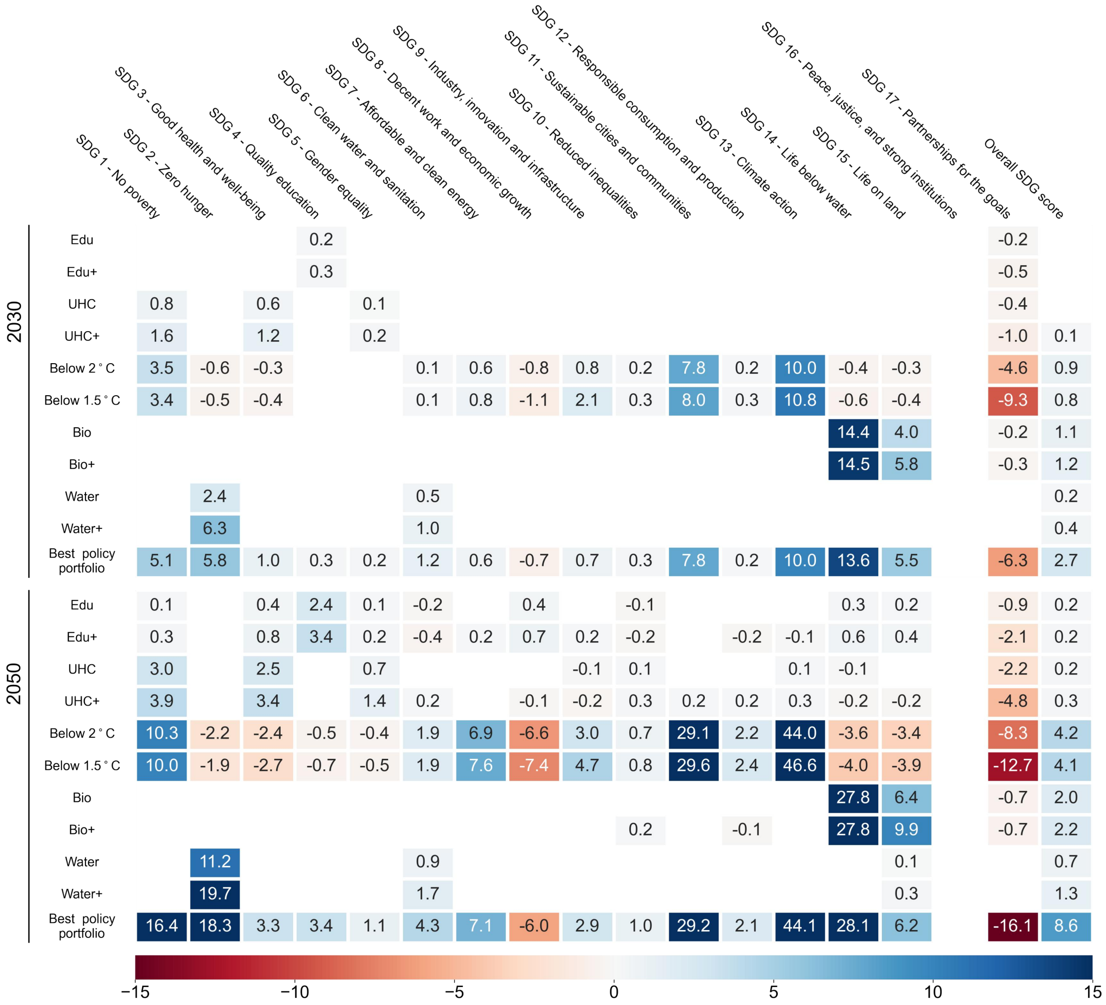

Supplementary Figure 8. Synergies and trade-offs of single-policy portfolios and the best policy portfolio (Edu+, UHC+, Below 2°C, Bio+ and Water+) compared to the baseline policy portfolio under the states of the world with climate change by 2030 and 2050. The coloured shading indicates under the states of the world with climate change, their performance differences as compared the baseline policy portfolio in individual SDG scores and overall SDG scores. Cells are blank where the differences were small (i.e., between -0.1 and 0.1).

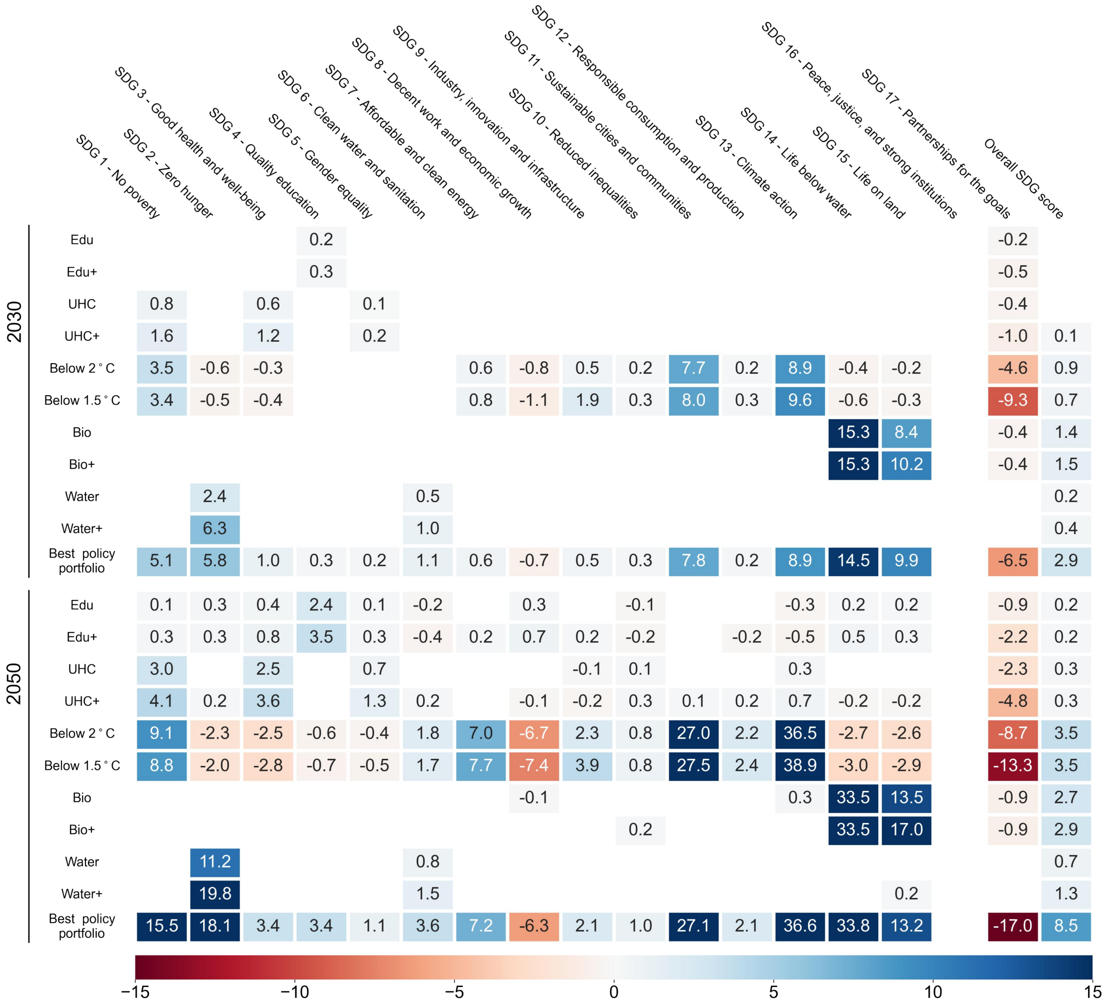

Supplementary Figure 9. Synergies and trade-offs of single-policy portfolios and the best policy portfolio (Edu+, UHC+, Below 2°C, Bio+ and Water+) compared to the baseline policy portfolio under the states of the world with biodiversity loss by 2030 and 2050. The coloured shading indicates under the states of the world with biodiversity loss, their performance differences as compared the baseline policy portfolio in individual SDG scores and overall SDG scores. Cells are blank where the differences were small (i.e., between -0.1 and 0.1).

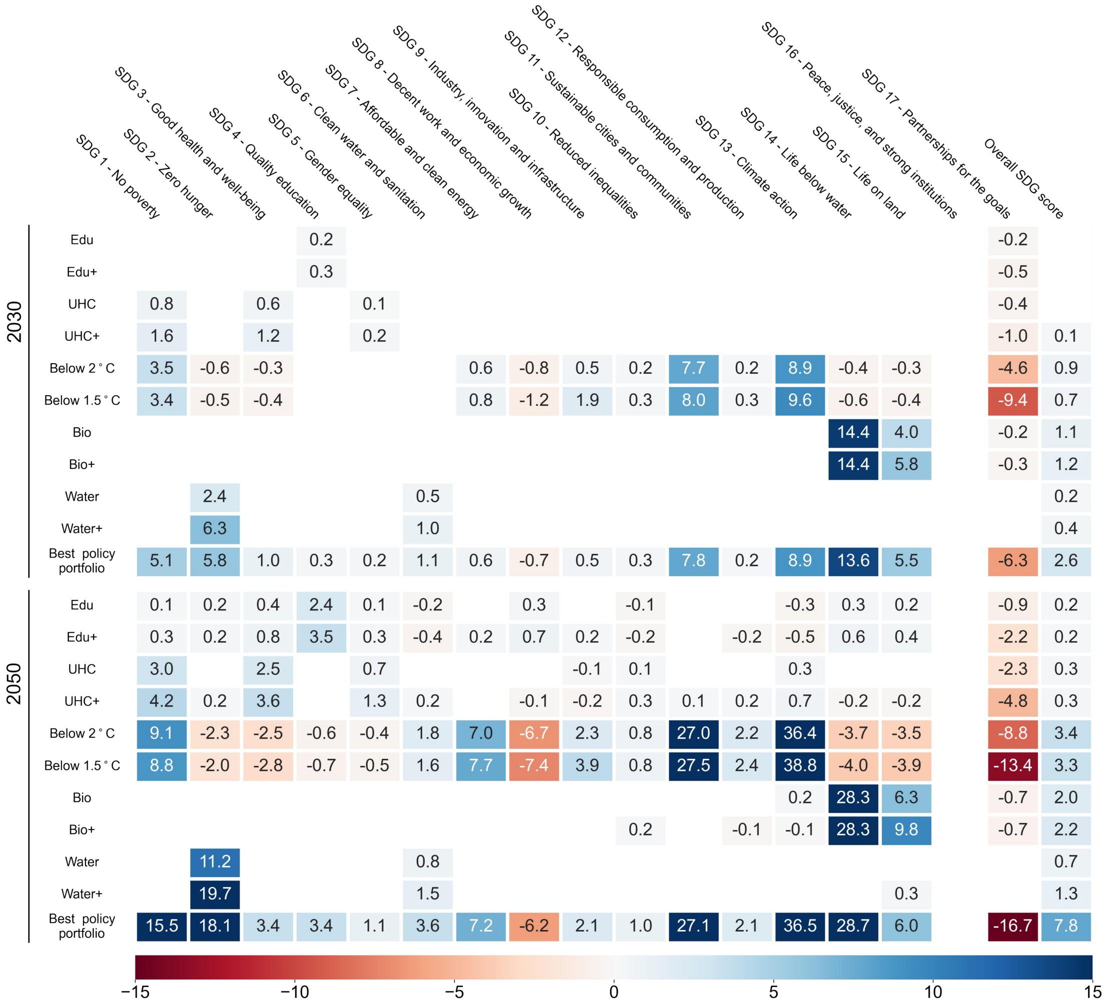

Supplementary Figure 10. Synergies and trade-offs of single-policy portfolios and the best policy portfolio (Edu+, UHC+, Below 2°C, Bio+ and Water+) compared to the baseline policy portfolio under the states of the world with no disruptors by 2030 and 2050. The coloured shading indicates under the states of the world with no disruptors, their performance differences as compared the baseline policy portfolio in individual SDG scores and overall SDG scores. Cells are blank where the differences were small (i.e., between -0.1 and 0.1).

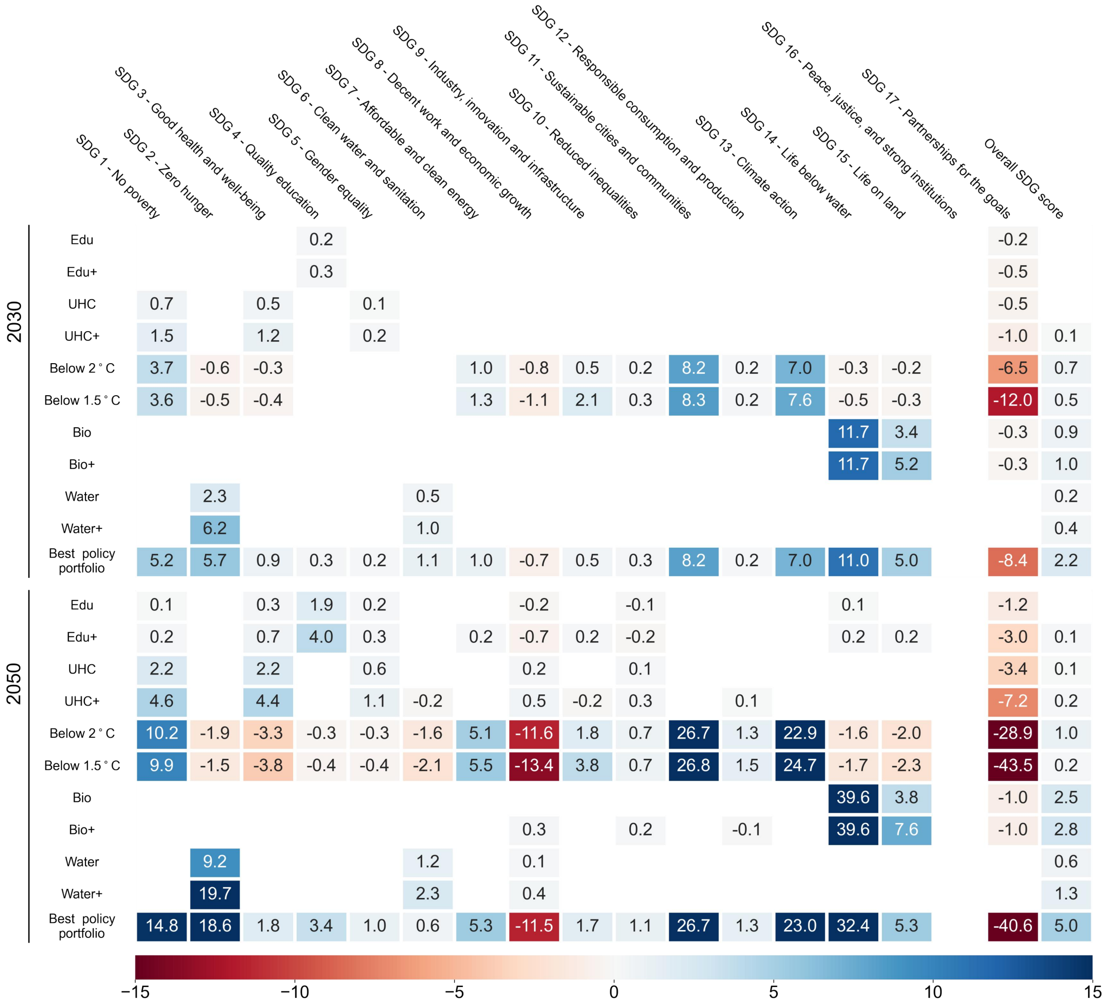

Supplementary Figure 11. Synergies and trade-offs of single-policy portfolios and the best policy portfolio (Edu+, UHC+, Below 2°C, Bio+ and Water+) compared to the baseline policy portfolio under the states of the world with all disruptors by 2030 and 2050. The coloured shading indicates under the states of the world with all disruptors, their performance differences as compared the baseline policy portfolio in individual SDG scores and overall SDG scores. Cells are blank where the differences were small (i.e., between -0.1 and 0.1).

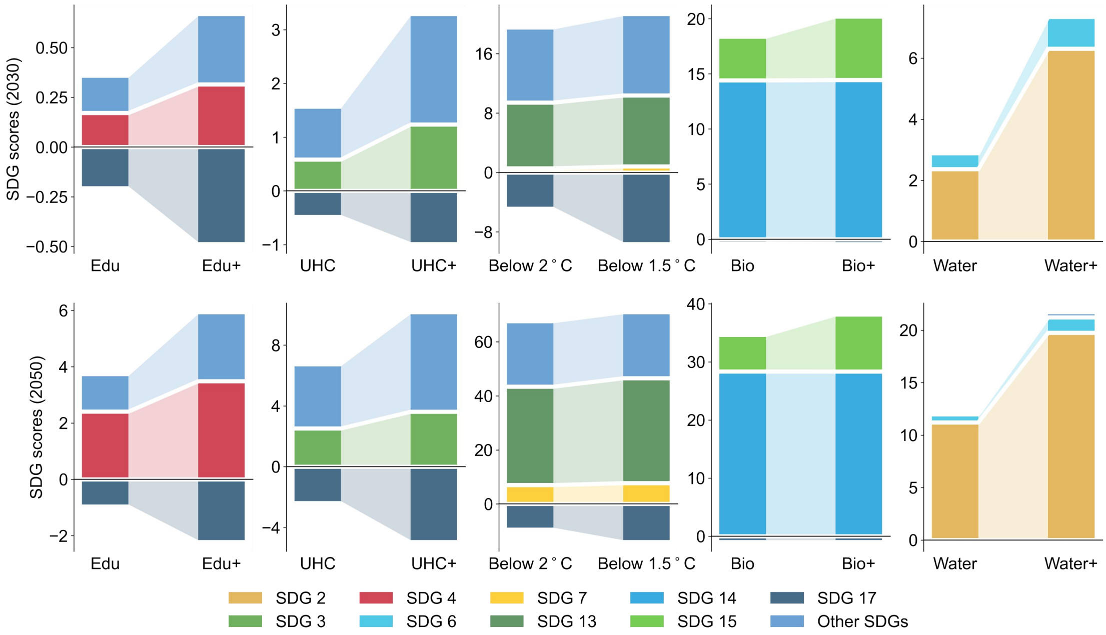

Supplementary Figure 12. Synergies and trade-offs of single-policy portfolios compared to the baseline policy portfolio under the states of the world with no disruptors by 2030 and 2050. In each plot, each bar indicates single-policy portfolio performance differences as compared against the baseline policy portfolio in related SDGs by 2030 or 2050.

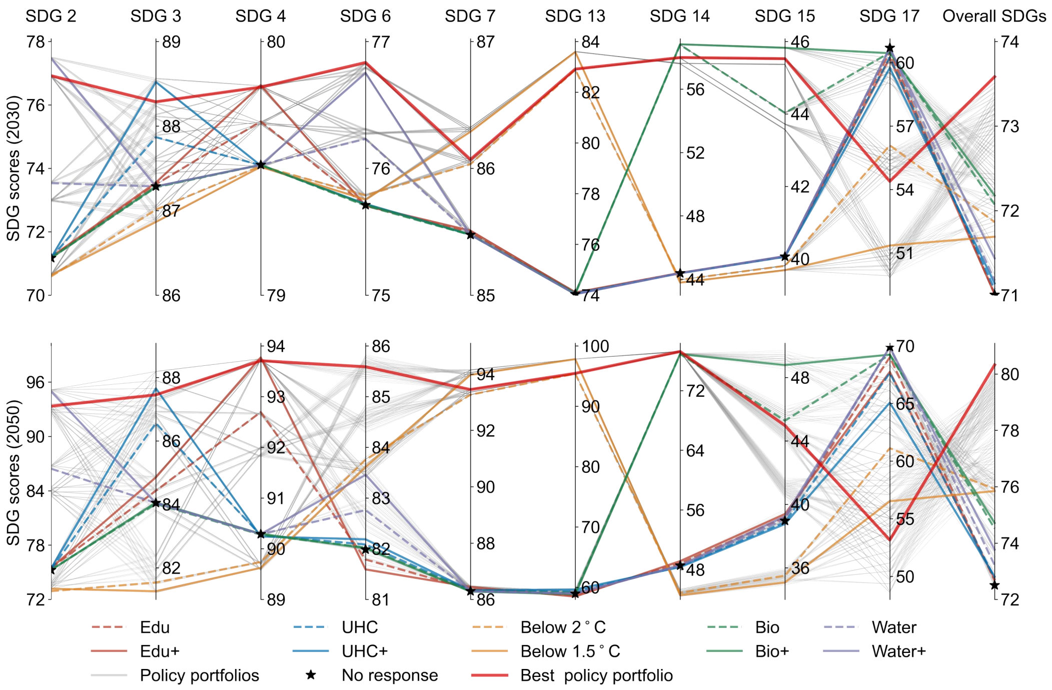

Supplementary Figure 13. Individual SDG scores and overall SDG scores of the policy portfolios under the baseline state of the world with no disruptors by 2030 and 2050. In each plot, each line indicates the average of individual SDG scores and overall SDG scores under the baseline state of the world with no disruptors. In particular, the star symbol denotes the scores of the baseline policy portfolio, the red solid line represents the scores of the best policy portfolio (Edu+, UHC+, Below 2℃, Bio+ and Water+), and the other coloured lines represent the scores of the moderate (dashed line) and ambitious (solid line) single-policy portfolios.

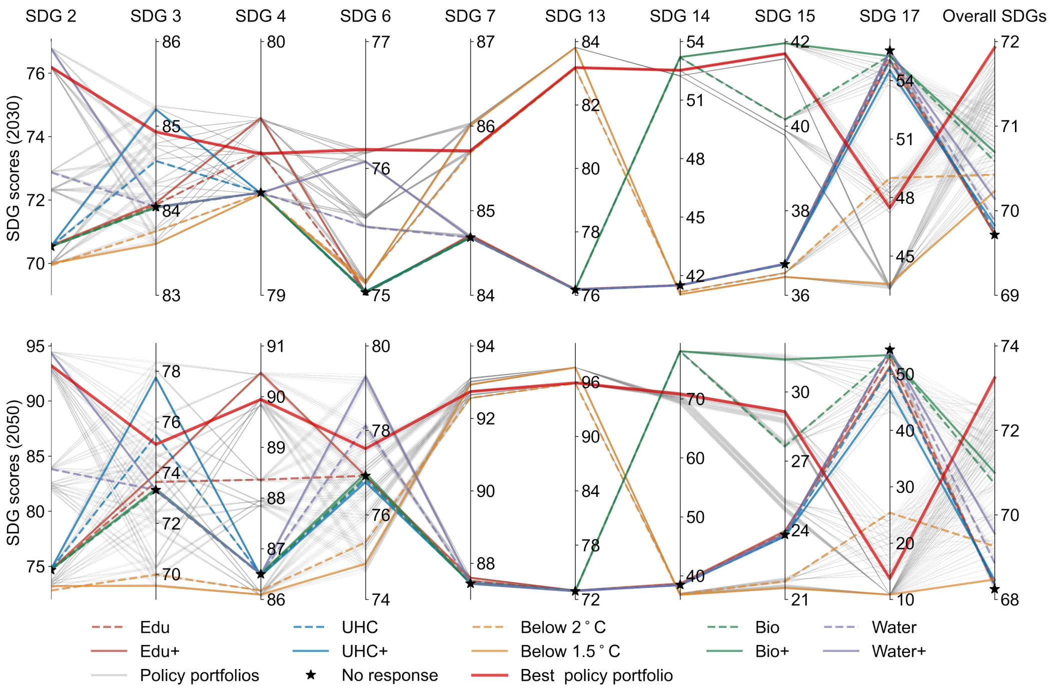

Supplementary Figure 14. Individual SDG scores and overall SDG scores of the policy portfolios under states of the world with all disruptors in 2030 and 2050. In each plot, each line indicates the average of individual SDG scores and overall SDG scores under 500 states of the world with all disruptors. In particular, the star symbol denotes the scores of the baseline policy portfolio, the red solid line represents the scores of the best policy portfolio (Edu+, UHC+, below 2℃, Bio+ and Water+), and the other coloured lines represent the scores of the moderate (dashed line) and ambitious (solid line) single-policy portfolios.

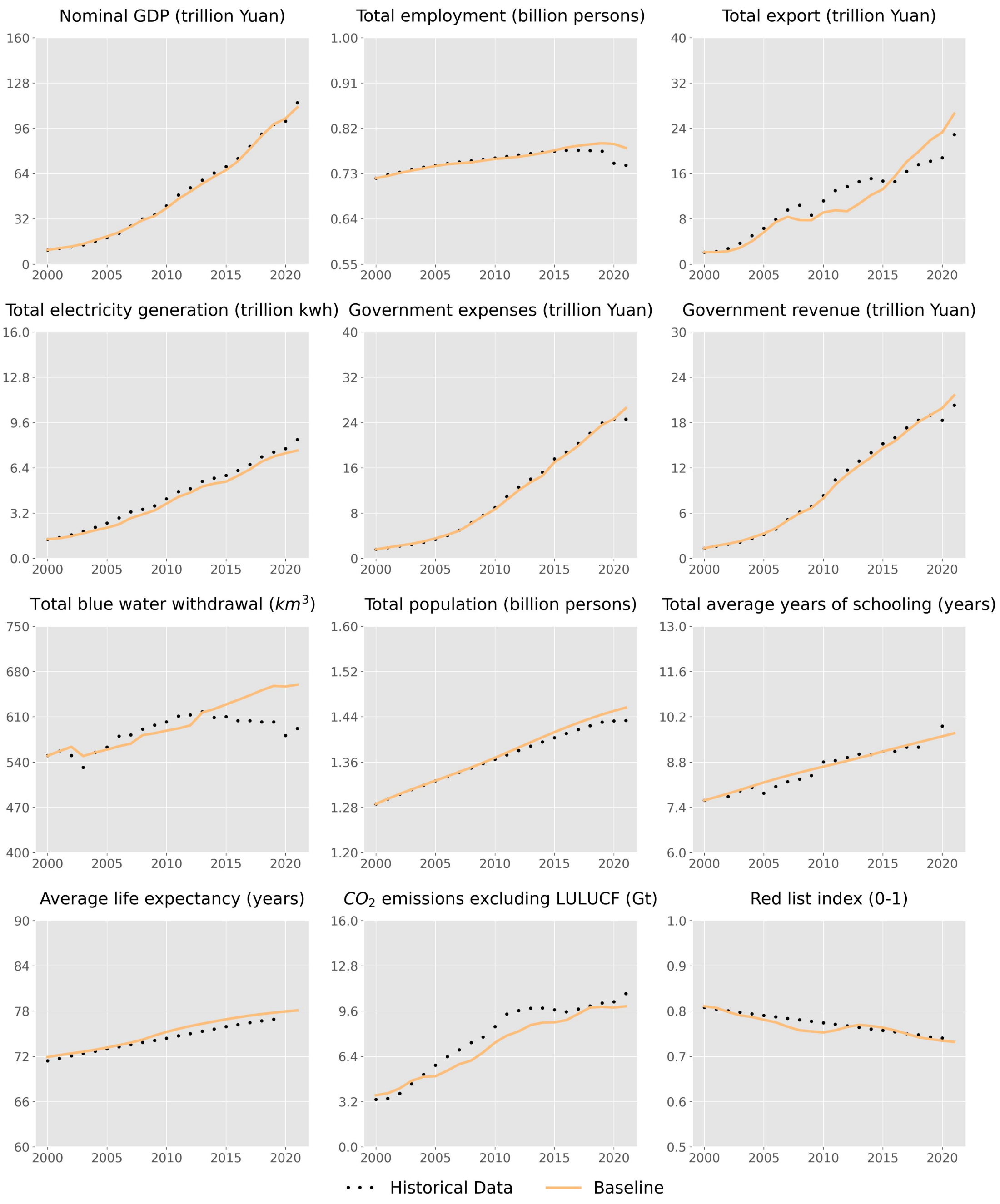

Supplementary Figure 15. Performances of a selection of variables in the iSDG-China model calibration.

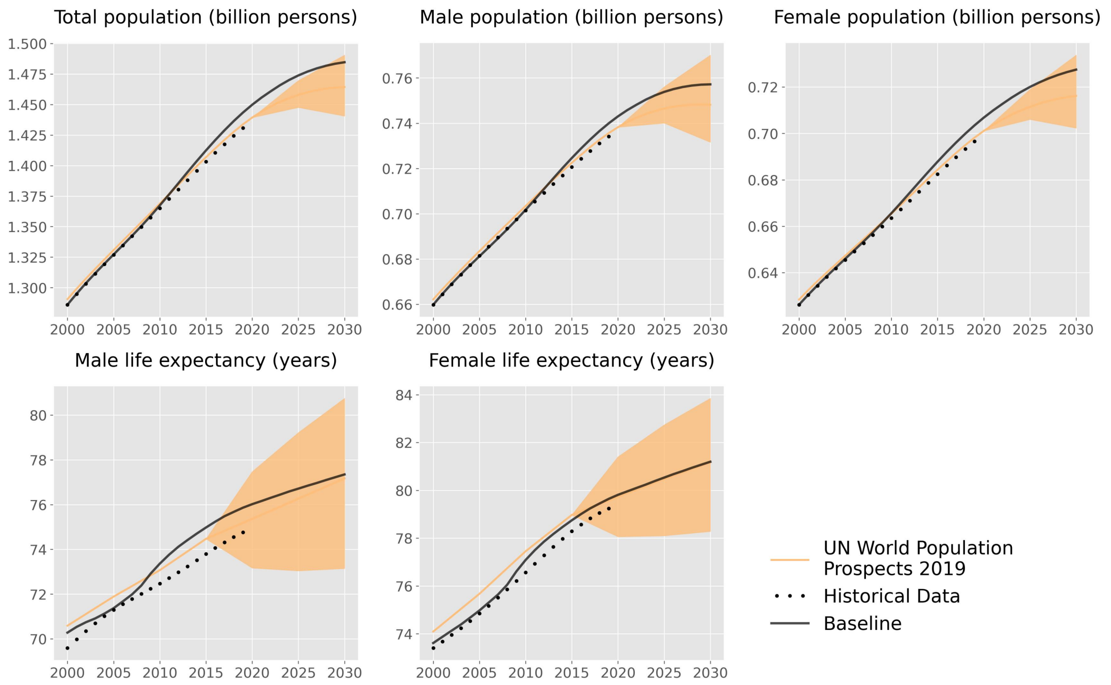

Supplementary Figure 16. The demographics of China projected by the iSDG-China model and the comparison against the United Nations World Population Prospects.

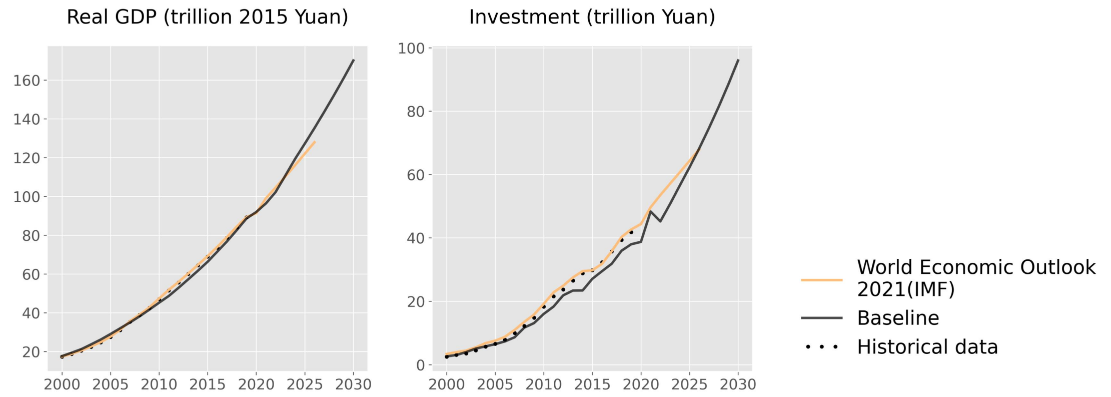

Supplementary Figure 17. The economic conditions of China projected by the iSDG-China model and the comparison against the World Economic Outlook.

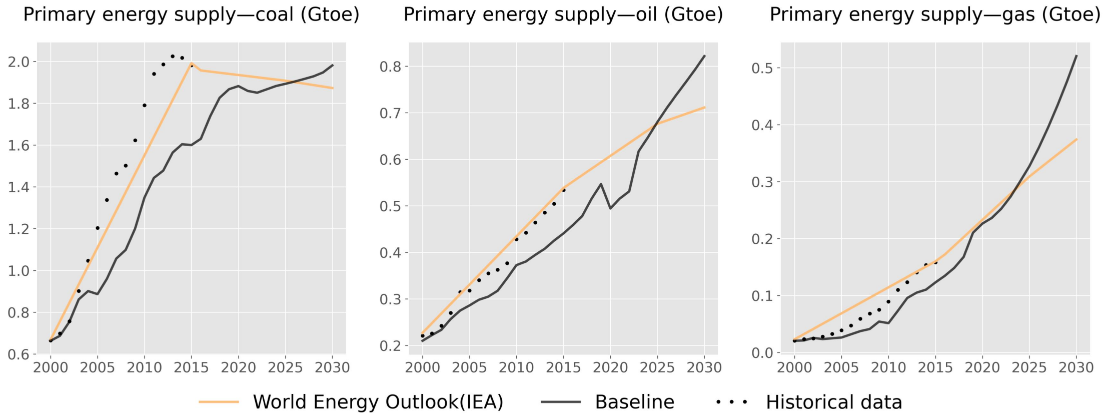

Supplementary Figure 18. The fossil energy consumption of China projected by the iSDG-China model and the comparison against the World Energy Outlook.

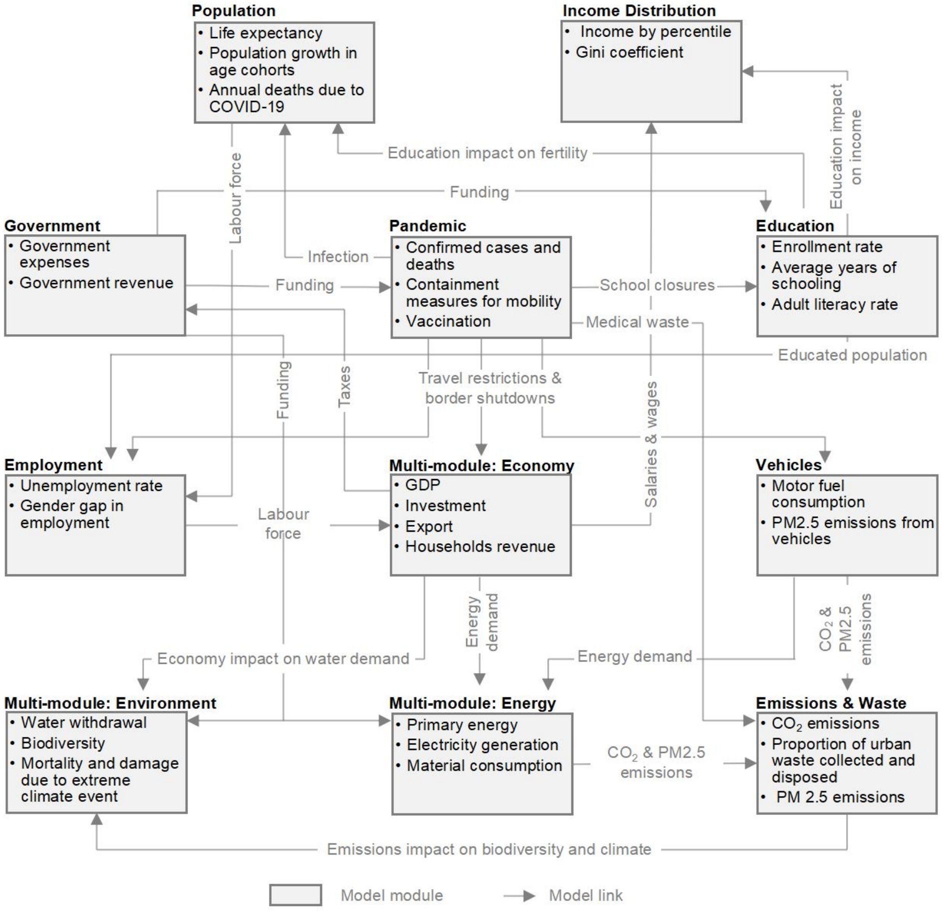

Supplementary Figure 19. Conceptual relationships between the pandemic module and other modules. The grey shaded boxes indicate different modules in SDG-China model and each arrow line connecting two modules indicates linkages between the two modules.

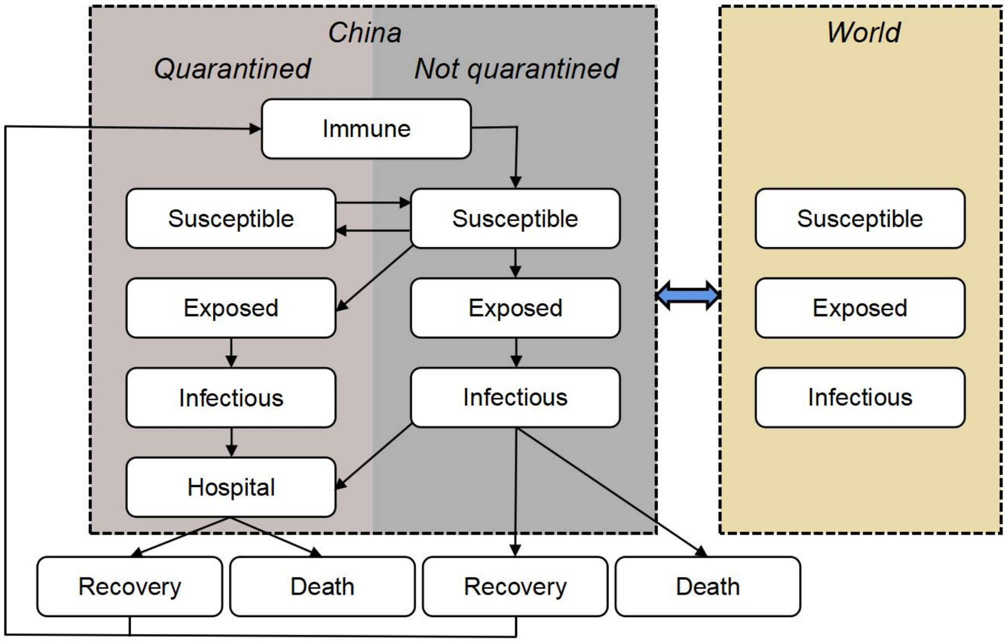

Supplementary Figure 20. The model structure of pandemic module.

Supplementary Tables

Supplementary Table 1. Summary of main parameters and associated quantitative severity of the identified disruptors.

Disruptors	Associated parameters	Severity	Source
**Pandemic disease**: Emerging and re-emerging infectious disease pandemics, resulting in serious health crisis and economic recession.	Transmission rate of pandemic disease	0－0.039	The upper bound of the severity is set according to the historical trend of the COVID-19 pandemic (calibrated based on data from 2020 to 2022).
	Infection-fatality rate of pandemic disease	0.07%－0.21%	The upper bound of the severity is set according to the historical trend (calibrated based on data from 2020 to 2022); and the lower bound is set based to the annual mortality burden of seasonal influenza[58].
**Ageing and shrinking population**: Demographic transition to an aging and shrinking population, which leads to shrinking labour force and requires more healthcare expenditure to support the needs of older adults.	Total fertility rate	1.05－1.45	Total fertility rate data form National Bureau of Statistics, national census data for China 2000-2020. The lower and upper bounds are set according to the low and medium fertility estimates[59] on the basis of national census data.
	Reference health expenditure requirement per capita ($)	2656–3345	The lower bound of the severity is set based on the historical trend (calibrated based on data for the universal health coverage[60] from 2000 to 2019). The upper bound is set as the upper bound of the uncertainty interval of China's health expenditure per capita (2282 in 2040 and 3.9% growth per annum from 2040 to 2050) in the projections of national health expenditure[61].
	Elasticity variation of productivity to ageing	Industry: -0.38－-0.15<br>Services: -0.38－-0.15	Uncertainty variation is estimated based on the GDP projection[62] of one-child policy under SSP2, in which China's GDP would reach 200 trillion Yuan by 2050.
**Deglobalization**: Deglobalization restricts access to foreign goods and services and adversely affects economic interactions between countries. Most countries would face high tariff, financial constraints, and shrinking foreign trade and capital flows.	Reference interest rate on foreign debt	1.5%－3%	Data from World Development Indicators[63] for China. The upper bound is set based on high historical values; and the lower bound is set based on World Development Indicators for 2016.
	Tax rate on international trade (% import)	2%－3%	Data from World Development Indicators[63] and Finance Yearbook of China[64]. The upper bound is set based on high historical values; and the lower bound is set based on World Development Indicators and Finance Yearbook of China for 2019.

	Capital elasticity variation	Industry:0.58－0.65<br>Services:0.56－0.63	The lower bound of the severity is set based on the historical trend (calibrated based on GDP from World Development Indicators[63] from 2000 to 2021). The upper bound is estimated based on the GDP projection[62] of two-child policy under SSP3, in which China's GDP would reach 180 trillion Yuan by 2050.
**Biodiversity loss**: Lack of long-term attention and adequate financial support, combined with increasing resource demand and more environmental pollution associated with industrialization and economic activities, leads to deforestation, terrestrial and marine ecosystem degradation, and reduced biodiversity.	Marine and terrestrial areas protection expenditure (% GDP)	0.12%－0.23%	Data from Finance Yearbook of China[64]. The upper bound is set based on Finance Yearbook of China for 2019; and the low bound is set based on low historical values.
	Afforestation area (Mha)	0	No additional afforestation.
**Climate change**: Trade-offs between socioeconomic development and environmental impacts diverts policy attention and resources away from climate action, resulting in higher energy and material consumption, slow progress on water-use efficiency, rising global temperatures, and an increase in the frequency and severity of extreme weather events.	Expected global energy intensity change per annum	0－1.5%	The upper bound is estimated based on World Development Indicators[63].
	Global average yearly change in material consumption per unit output	0－0.775%	Estimated based on International Resource Panel[65] and Sustainable Europe Research Institute[66].
	Relative average global water efficiency (compared to year 2000)	1.46－1.825	The upper bound is estimated based on Development Indicators[63] and OECD environmental outlook[67]; and the lower bound is sets as a 20% reduction on the upper bound.
	Climate change temperature (above pre-industrial)	1.734℃－2.211℃	The lower and upper bounds are set based on the RCP4.5 and RCP8.5 scenarios[68], respectively.

Supplementary Table 2. The descriptions and sources of policies from the five policy clusters.

Policy Clusters	Policies	Descriptions	Sources
Education	No response	Enrolment rates for primary secondary and tertiary education increase with historical trends, achieving 100%, 100% and 55% by 2050, respectively.	Calibrated based on data from World Development Indicators[63] for China from 2000 to 2021. We assume that the government expenditure on education as a share of GDP remains unchanged at 3.5%[64] since 2021.
	Edu	Enrolment rates for primary secondary and tertiary education achieve 100%, 100% and 65% by 2050, respectively.	We assume that the enrolment rates for tertiary education reaches 65% by 2050, which is 15 years later than China's education modernization 2035[69]. And the education expenditure as a share of GDP reaches 3.8% by 2050.
	Edu+	Enrolment rates for primary secondary and tertiary education achieve 100%, 100% and 80% by 2050, respectively.	According to China's education modernization 2035[69], the enrolment rates for tertiary education reaches 65% by 2035. According to this trend, the enrolment rates for tertiary education reaches 80% by 2050. The education expenditure as a share of GDP is at 4.2% by 2050.
Health	No response	Government health expenditure as a share of GDP follows historical trends.	Data of government expenditure on health is from Finance Yearbook of China[64]. We assume that the government expenditure on health as a share of GDP remains unchanged at 1.7% since 2021.
	UHC	By 2050, the universal health coverage (UHC) achieves 100%.	We assume that SDGs 3.8.1 (i.e., all people have access to the full range of quality health services) achieves by 2050 under this policy. The government expenditure on health as a share of GDP reaches 2.3% by 2050.
	UHC+	By 2030, the UHC achieves 100%.	The UHC+ is set as specified in the SDGs 3.8.1. We assume that the government expenditure on health as a share of GDP reaches 3% by 2050.
Energy	No response	By 2050, power generated from non-fossil energy and energy efficiency continues historical trends.	Calibrated based on data from World Development Indicators[63] for China from 2000 to 2021.
	Below 2°C	By 2050, power generated from non-fossil energy reaches 90.5% and CO_2 emissions per unit of energy consumption reaches 0.56 $kgCO_2\ kgce^{-1}$.	Parameterized based on the below 2°C scenario[70].
	Below 1.5°C	By 2050, power generated from non-fossil energy reaches 91.2% and CO_2 emissions per unit of energy consumption reaches 0.29 $kgCO_2\ kgce^{-1}$.	Parameterized based on the below1.5°C scenario[70].
Water	No response	The share of harvested area irrigated and agriculture water-use efficiency follow historical trend.	Agriculture water efficiency is estimated based on World Development Indicators[63] and OECD environmental outlook[67]. By 2050, the irrigation water withdrawal is projected to reach 290 billion cubic meters.
	Water	A 15% increase in the share of harvested area under efficient irrigation and sustainable management compared to the baseline	Parameterized based on 15% improvement on the no response policy. Under the Water policy, the harvested area under efficient irrigation increases by 7.9 Mha and the irrigation water withdrawal

		policy by 2050.	reaches 273 billion cubic meters.
	Water+	A 30% increase in the share of harvested area under efficient irrigation and sustainable management compared to the baseline policy by 2050.	Parameterized based on 30% improvement on the no response policy. Under the Water+ policy, the harvested area under efficient irrigation increases by 15.8 Mha and the irrigation water withdrawal reaches 258 billion cubic meters.
Land use	No response	Terrestrial and marine areas effectively protected follow historical trends and no increase in afforestation.	The government expenditure on terrestrial and marine areas is from Finance Yearbook of China[64]; Forest cover is calibrated based on data from China Forestry and Grassland Statistical Yearbook[71].
	Bio	By 2050 at least 30% of terrestrial and marine areas are effectively conserved and no increase in afforestation.	According to Kunming-Montreal Global Biodiversity Framework[72], at least 30% of areas of degraded terrestrial, inland water, and coastal and marine ecosystems are under effective restoration.
	Bio+	By 2050, at least 30% of terrestrial and marine areas are effectively conserved and afforestation area gradually reaches 31.8 Mha.	The terrestrial and marine areas effectively protected is based on Kunming-Montreal Global Biodiversity Framework[72], and the additional afforestation after 2020 is set to as the maximum potential for China (31.8 Mha)[73].

Supplementary Table 3. Summary of main drivers and associated qualitative and quantitative assumptions and policy settings of the Baseline scenario.

Drivers	Assumption /Policy	Attributes	Value	Source
Demographics	Assumption	Population growth	Medium	Qualitative. SSP narratives[74].
	Assumption	Fertility	1.72 by 2030; 1.75 by 2050	Parameterized based on China's historical values of World Population Prospects[75].
	Assumption	Mortality	Medium	Qualitative. SSP narratives[74].
	Assumption	Migration	Medium	Qualitative. SSP narratives[74].
	Assumption	Urbanization	0.706 by 2030; 0.8 by 2050	Urbanization data from World Urbanization Prospects: The 2018 Revision[14].
	Policy	Annual net migration (per 1,000 population)	-0.241 by 2030; -0.219 by 2050	Parameterized based on China's historical values of World Population Prospects[75].
Human development	Assumption	Education	Medium	Qualitative. SSP narratives[74].
	Assumption	Access to health	Medium	Qualitative. SSP narratives[74].
	Assumption	Gender equality	Medium	Qualitative. SSP narratives[74].
	Assumption	Social cohesion	Medium	Qualitative. SSP narratives[74].
	Assumption	Societal participation	Medium	Qualitative. SSP narratives[74].
	Policy	Education expenditure (% GDP)	3.5	Education expenditure data from Finance Yearbook of China[67].
	Policy	Health expenditure (% GDP)	1.7	Health expenditure data from Finance Yearbook of China[67].
	Assumption	Gender－Target effect of Social & Market framework	0.06	Parameterized based on iSDG model settings[56,76].
	Assumption	Political stability (Score -2.5 to +2.5)	-0.261	Data from Worldwide Governance Indicator[77] for China.
	Assumption	Voice and accountability (Score -2.5 to +2.5)	-1.449	Data from Worldwide Governance indicator[77] for China.
	Policy	Subsidies & transfers (% GDP)	3.66	Subsidies and transfers expenditure data from Finance Yearbook of China[64].
Economy and lifestyle	Assumption	Growth	Medium	Qualitative. SSP narratives[74].
	Assumption	Inequality	Uneven moderate reductions within countries	Qualitative. SSP narratives[74].
	Assumption	International trade	Moderate	Qualitative. SSP narratives[74].
	Assumption	Globalization	Semi-open globalized economy	Qualitative. SSP narratives[74].
	Assumption	Consumption and diet	Material-intensive consumption, medium meat consumption	Qualitative. SSP narratives[74].

	Assumption	Demand for resources exports and consumption: Domestic material consumption per capita (ton)	87	Calibrated based on data from Material consumption indicator[78] for China.
	Assumption	Demand for agriculture exports & consumption: Crops ($ 2015 per ton)	Cereals:379, Rest: 480	Data for China from FAOSTAT database[79].
	Assumption	Demand for agriculture exports & consumption: Livestock ($ 2015 per ton)	1896	Data for China from FAOSTAT database[79].
	Assumption	Demand for agriculture exports & consumption: Forest ($ 2015 per cubic meter)	107	Data for China from FAOSTAT database[79].
	Assumption	Interest rates on debt (%)	1.5	Data from World Development Indicators[63] and Finance Yearbook of China[64].
	Policy	Tax rate on international trade (% imports)	2.0	Data from World Development Indicators[63] and Finance Yearbook of China[64].
	Policy	Infrastructure expenditure (% GDP)	1.12	Data from Finance Yearbook of China[64].
	Policy	Tax rate on income and profits (% GDP)	8.5	Data from Finance Yearbook of China[64]; Based on a 20% improvement compared to 2021, to improve domestic capacity for tax.
	Policy	Tax rate on goods and services (% GDP)	10.9	Data from Finance Yearbook of China[64]; Based on a 20% improvement compared to 2021, to improve domestic capacity for tax.
Environment & natural resources	Assumption	Fossil constraints	No reluctance to use unconventional resources	Qualitative. SSP narratives[74].
	Assumption	Environment	Continued degradation	Qualitative. SSP narratives[74].
	Assumption	Land use	Medium regulations lead to slow decline in the rate of deforestation	Qualitative. SSP narratives[74].
	Assumption	Agriculture	Medium pace of tech change in ag sector; entry barriers to ag markets reduced slowly	Qualitative. SSP narratives[74].
	Policy	Expenditure for protected areas (% GDP)	0.23	Data from Finance Yearbook of China[64].
Technology	Assumption	Development & Change	Medium	Qualitative. SSP narratives[74].
	Assumption	Carbon intensity	Medium	Qualitative. SSP narratives[74].
	Assumption	Energy intensity	Medium	Qualitative. SSP narratives[74].
	Assumption	Climate change temperature (above pre-industrial)	1.13°C by 2030 and 1.734°C by 2050	Average temperature estimated based on the RCP4.5 scenario[68].

	Assumption	Expected global energy intensity change per annum (%)	-1.5	Expected energy intensity changes estimated based on World Development Indicators[63].
	Assumption	Global average yearly change in material consumption per unit output (%)	-0.775	Material efficiency changes estimated based on International Resource Panel[65] and Sustainable Europe Research Institute[66].
	Assumption	Relative average global water efficiency (compared to year 2000)	1.825	Global water efficiency change estimated based on World Development Indicators[63] and OECD environmental outlook[67].
	Policy	Target material consumption efficiency improvement	None	Based on iSDG model setting[56,76].
	Policy	SDG Climate change–adaptation expenditure	None	SDG adaptation expenditure based on iSDG model projections of adaptation costs.
Governance, policy and institutions	Assumption	Institutions	Modest effectiveness	Qualitative. SSP narratives[74].
	Assumption	International cooperation	Relatively weak	Qualitative. SSP narratives[74].
	Assumption	Environmental policy	Concern for local pollutants but only moderate success in implementation	Qualitative. SSP narratives[74].
	Assumption	Policy orientation	Weak focus on sustainability	Qualitative. SSP narratives[74].
	Policy	Administrative expenditure (% GDP)	11.48	Data from Finance Yearbook of China[64].
	Policy	Government expenditure salaries & wages (% consumption)	50.7	Data from China Statistical Yearbook[15].
	Assumption	Government effectiveness (Score -2.5 to +2.5)	0.477	Data from World Bank Worldwide Governance Indicator[77] for China.
	Assumption	Control of corruption (Score -2.5 to +2.5)	-0.271	Data from World Bank Worldwide Governance Indicator[77] for China.
	Assumption	Rule of law (Score -2.5 to +2.5)	-0.202	Data from World Bank Worldwide Governance Indicator[77] for China.
	Assumption	Regulatory quality (Score -2.5 to +2.5)	-0.135	Data from World Bank Worldwide Governance Indicator[77] for China.

Supplementary Table 4. Upper and lower bounds of SDG indicators for assessing SDG implementation in the iSDG-China model.

Goal	Target	Indicator	D#	Value		Source	
				Upper bound	Lower bound	Upper bound	Lower bound
SDG 1. No Poverty	1.1 By 2030, eradicate extreme poverty for all people everywhere, currently measured as people living on less than $1.25 a day	010101. Proportion of population below poverty line (%)	Ec	0	72.6	SDG Target 1.1 is to eradicate extreme poverty.	SDG Index and Dashboards Report[80].
	1.2 By 2030, reduce at least by half the proportion of men, women and children of all ages living in poverty in all its dimensions according to national definitions	010201. Proportion of population below national poverty line (%)	Ec	0	63.8	SDG Target 1.2 is to halve baseline value.	Bottom 2.5th-percentile prformer[63].
	1.4 By 2030, ensure that all men and women, in particular the poor and the vulnerable, have equal rights to economic resources, as well as access to basic services, ownership and control over land and other forms of property, inheritance, natural resources, appropriate new technology and financial services, including microfinance	010401. Average access to basic health care (worst 0–1 best)	S	1	0.382	Leave no one behind. SDG Target 1.4 already achieved for China.	SDG Index and Dashboards Report[80].
	1.5 By 2030, build the resilience of the poor and those in vulnerable situations and reduce their exposure and vulnerability to climate-related extreme events and other economic, social and environmental shocks and disasters	010501a. Mortality due to disasters five year (per 100,000 population, 5-year average)	S	0.2745	1.476	Sendai Framework DRR targets for 2030 are to "substantially reduce"[81]; target sets as 50% improvement on baseline value.	China Statistical Yearbook[15]; China Statistical Yearbook on Environment[82].
		010501b. Proportion of population affected by natural disasters (%, 5-year average)	S	5.658	32.08	Sendai Framework DRR targets for 2030 are to "substantially reduce"[81]; target sets as 50% improvement on baseline value.	China Statistical Yearbook[15]; China Statistical Yearbook on Environment[82].
		010502. Economic damage due to natural disasters (% of GDP, 5-year average)	Ec	0.39635	1.577	Sendai Framework DRR targets for 2030 are to "substantially reduce"[81];	China Statistical Yearbook[15]; China Statistical Yearbook on

						target sets as 50% improvement on baseline value.	Environment[82].
SDG 2. Zero Hunger	2.1 By 2030, end hunger and ensure access by all people, in particular the poor and people in vulnerable situations, including infants, to safe, nutritious and sufficient food all year round	020101. Prevalence of undernourishment (%)	S	0	42.3	SDG Target 2.1 is to "end hunger".	SDG Index and Dashboards Report[80].
	2.2 By 2030, end all forms of malnutrition, including achieving, by 2025, the internationally agreed targets on stunting and wasting in children under 5 years of age, and address the nutritional needs of adolescent girls, pregnant and lactating women and older persons	020201. Prevalence of stunting (%)	S	0	50.2	SDG Target 2.2 end all forms of malnutrition.	SDG Index and Dashboards Report[80].
		020202. Prevalence of malnutrition (%)	S	0	16.3	SDG Target 2.2 end all forms of malnutrition.	SDG Index and Dashboards Report[80].
	2.3 By 2030, double the agricultural productivity and incomes of small-scale food producers, in particular women, indigenous peoples, family farmers, pastoralists and fishers, including through secure and equal access to land, other productive resources and inputs, knowledge, financial services, markets and opportunities for value addition and non-farm employment	020301. Total agriculture production per labor unit (t/person)	Ec	16.657	3.464	SDG Target 2.3 is to double productivity compared to baseline value.	Baseline projection.
		020301Alt. Cereal yield (t/ha)	Ec	7	0.2	SDG Index and Dashboards Report[80].	SDG Index and Dashboards Report[80].
	2.4 By 2030, ensure sustainable food production systems and implement resilient agricultural practices that increase productivity and production, that help maintain ecosystems, that strengthen capacity for adaptation to climate change, extreme weather, drought, flooding and other disasters and that progressively improve land and soil quality	020401. Proportion of harvested area sustainably managed (% of harvested area)	En	22.58	0	Average of 5 best performers[79].	Bottom 2.5th-percentile prformer[79].
SDG 3. Good Health and Well-being	3.1 By 2030, reduce the global maternal mortality ratio to less than 70 per 100,000 live births	030101. Maternal mortality ratio (per 100,000 population)	S	0	814	Leave no one behind.	SDG Index and Dashboards Report[80].
		030102. Average access to basic health care (worst 0–1 best)	S	1	0.382	Leave no one behind.	SDG Index and Dashboards Report[80].

	3.2 By 2030, end preventable deaths of newborns and children under 5 years of age, with all countries aiming to reduce neonatal mortality to at least as low as 12 per 1,000 live births and under-5 mortality to at least as low as 25 per 1,000 live births	030201. Under five mortality rate (per 1,000 population)	S	0	130.1	Leave no one behind.	SDG Index and Dashboards Report[80].
		030202. Neonatal mortality rate (per 1,000 population)	S	0	39.7	Leave no one behind.	SDG Index and Dashboards Report[80].
	3.3 By 2030, end the epidemics of AIDS, tuberculosis, malaria and neglected tropical diseases and combat hepatitis, water-borne diseases and other communicable diseases	030301New. Annual deaths due to COVID-19 (per 100,000 population)	S	0	355.319	Average of 5 best performers[3].	Bottom 2.5th-percentile prformer[3].
	3.4 By 2030, reduce by one third premature mortality from non-communicable diseases through prevention and treatment and promote mental health and well being	030401. Cardiovascular neoplasm diabetes and respiratory mortality (per 1,000 population)	S	2.549	9.537	SDG Target 3.4 is to reduce by 1/3 on baseline value.	Bottom 2.5th-percentile prformer[83].
	3.6 By 2020, halve the number of global deaths and injuries from road traffic accidents	030601. Total mortality rates by cause -road (per 1,000 population)	S	0.0889	0.4156	SDG Target 3.6 is to halve baseline value.	Bottom 2.5th-percentile prformer[84].
	3.7 By 2030, ensure universal access to sexual and reproductive health-care services, including for family planning, information and education, and the integration of reproductive health into national strategies and programmes	030701. Contraceptive prevalence rate (%)	S	100	17.5	Leave no one behind.	SDG Index and Dashboards Report[80].
		030702. Adolescent birth rate (per 1,000 population)	S	2.5	139.6	SDG Index and Dashboards Report[80].	SDG Index and Dashboards Report[80].
	3.8 Achieve universal health coverage, including financial risk protection, access to quality essential health-care services and access to safe, effective, quality and affordable essential medicines and vaccines for all	030801. Average access to basic health care (worst 0–1 best)	S	1	0.382	Leave no one behind. SDG Target 3.8 already achieved for China. Maintain performance	SDG Index and Dashboards Report[80].
		030801Alt. Life expectancy at birth (year)	S	83	54	SDG Index and Dashboards Report[80].	SDG Index and Dashboards Report[80].
SDG 4. Quality Education	4.1 By 2030, ensure that all girls and boys complete free, equitable and quality primary and secondary education leading to relevant and effective learning outcomes	040101. Proportion of population completed secondary school (% of population age 20 to 24)	S	90	28.15	SDG Target 4.1 is "universal completion". Target is set based on Allen et al.[76].	Baseline projection.
	4.3 By 2030, ensure equal access for all women and men to affordable and quality technical,	040301. Proportion of population enrolled in tertiary education (% of	S	52.2	0	SDG Index and Dashboards Report[80].	SDG Index and Dashboards Report[80].

	vocational and tertiary education, including university	population age 20 to 29)					
		040301Alt. Women proportion of total tertiary graduates (%)	S	50	32.8	SDG Target 4.3 is for "equal access" or gender parity.	Bottom 2.5th-percentile prformer[85].
	4.5 By 2030, eliminate gender disparities in education and ensure equal access to all levels of education and vocational training for the vulnerable, including persons with disabilities, indigenous peoples and children in vulnerable situations	040501. Adult literacy gender gap ratio (best 0－1 worst)	S	0	0.1121	SDG Target 4.5 is to "eliminate gender disparity".	Educational Statistics Yearbook of China[86].
	4.6 By 2030, ensure that all youth and a substantial proportion of adults, both men and women, achieve literacy and numeracy	040601. Average adult literacy rate (% of total population)	S	100	45.2	Leave no one behind. SDG Target 4.6 is a "substantial proportion" of adults. Target already achieved for China.	SDG Index and Dashboards Report[80].
		040601Alt. Total average years of schooling (year)	S	13.44	2.6	Average of 5 best performers[87].	Bottom 2.5th-percentile prformer[87].
SDG 5. Gender Equality	5.5 Ensure women's full and effective participation and equal opportunities for leadership at all levels of decision-making in political, economic and public life	050501. Female share of employment in managerial positions (%)	S	50	17.4	SDG Target 5.5 is for "equal opportunity".	Bottom 2.5th-percentile prformer[88].
		050501Alt. Employment to population ratio (female/male parity)	S	1	0.215	SDG Index and Dashboards Report[80].	SDG Index and Dashboards Report[80].
	5.6 Ensure universal access to sexual and reproductive health and reproductive rights as agreed in accordance with the Programme of Action of the International Conference on Population and Development and the Beijing Platform for Action and the outcome documents of their review conferences	050601. Contraceptive prevalence rate (%)	S	100	17.5	Leave no one behind.	SDG Index and Dashboards Report[80].
SDG 6. Clean Water and	6.1 By 2030, achieve universal and equitable access to safe and affordable drinking water for all	060101. Average access to safely managed water source (worst 0–1 best)	En	1	0.105	Leave no one behind.	SDG Index and Dashboards Report[80].

Sanitation	6.2 By 2030, achieve access to adequate and equitable sanitation and hygiene for all and end open defecation, paying special attention to the needs of women and girls and those in vulnerable situations	060201. Average access to safely managed sanitation facility (worst 0–1 best)	En	1	0.141	Leave no one behind.	SDG Index and Dashboards Report[80].
	6.4 By 2030, substantially increase water-use efficiency across all sectors and ensure sustainable withdrawals and supply of freshwater to address water scarcity and substantially reduce the number of people suffering from water scarcity	060402. Water resources vulnerability index (best 0–1 worst)	En	0.125	0.4	Target sets as Xu et al.[89].	A country is considered water scarce if annual withdrawals are between 20 and 40% of annual supply, and severely water scarce if withdrawals exceed 40%.
SDG 7. Affordable and Clean Energy	7.1 By 2030, ensure universal access to affordable, reliable and modern energy services	070101. Population with access to electricity (%)	S	100	9.1	Leave no one behind. SDG Target 7.1 already achieved for China.	SDG Index and Dashboards Report[80].
	7.2 By 2030, increase substantially the share of renewable energy in the global energy mix	070201. Share of renewable energy in total primary energy supply (%)	En	51	3	SDG Target 7.2 is to "increase substantially". Target sets as SDG Index and Dashboards Report[80].	SDG Index and Dashboards Report[80].
		070201Alt. Renewable share in electricity (%)	En	100	0	Average of 5 best performers[90].	Bottom 2.5th-percentile prformer[90].
		070202. CO2 emissions from fuel combustion for electricity per total electricity output (Mt/TWh)	En	0	5.9	SDG Index and Dashboards Report[80].	SDG Index and Dashboards Report[80].
	7.3 By 2030, double the global rate of improvement in energy efficiency	070301. Energy intensity level of primary energy (Mj/$ 2011)	En	3.159	16.56	SDG Target 7.3 is to double the rate of improvement.	Bottom 2.5th-percentile prformer[91].
SDG 8. Decent Work and Economic Growth	8.1 Sustain per capita economic growth in accordance with national circumstances and, in particular, at least 7 per cent gross domestic product growth per annum in the least developed countries	080101. Real GDP growth rate per capita (%)	Ec	5.5	-14.7	SDG Index and Dashboards Report[80].	SDG Index and Dashboards Report[80].
		080101. Real GDP per capita (2015 Yuan/person)	Ec	446792	3192	Target is set based on a real GDP growth rate of 5.5% (SDG Index and Dashboards Report[80]).	Bottom 2.5th-percentile prformer[92].
	8.2 Achieve higher levels of economic	080201. Real GDP per employed	Ec	715155	12699	Target is set based on a	Bottom 2.5th-percentile

	productivity through diversification, technological upgrading and innovation, including through a focus on high-value added and labour-intensive sectors	person (2015 Yuan/person)				growth rate of 8.24% (Average of 5 best performers[93]).	prformer[93].
	8.4 Improve progressively, through 2030, global resource efficiency in consumption and production and endeavour to decouple economic growth from environmental degradation, in accordance with the 10-Year Framework of Programmes on Sustainable Consumption and Production, with developed countries taking the lead	080401. Material footprint per unit of output (kg/$ 2011)	En	0.167	6.3252	Average of 5 best performers[94].	Bottom 2.5th-percentile prformer[94].
		080402. Domestic material consumption per unit of output (kg/$ 2011)	En	0.181	4.178	Average of 5 best performers[95].	Bottom 2.5th-percentile pformer[95].
	8.5 By 2030, achieve full and productive employment and decent work for all women and men, including for young people and persons with disabilities, and equal pay for work of equal value	080502. Unemployment rate (%)	Ec	0.5	25.9	SDG Target 8.5 is "full employment". Target sets as SDG Index and Dashboards Report[80].	SDG Index and Dashboards Report[80].
		080502Alt. Average employment to adult population ratio (%)	Ec	77.8	50	SDG Index and Dashboards Report[80].	SDG Index and Dashboards Report[80].
	8.6 By 2020, substantially reduce the proportion of youth not in employment, education or training	080601. Youth not in education employment or training (%)	S	8.1	28.2	SDG Index and Dashboards Report[80].	SDG Index and Dashboards Report[80].
SDG 9. Industry, Innovation and Infrastructure	9.1 Develop quality, reliable, sustainable and resilient infrastructure, including regional and transborder infrastructure, to support economic development and human well-being, with a focus on affordable and equitable access for all	090101. Rural access index (worst 0–1 best)	Ec	1	0.194	Leave no one behind. SDG Target 9.1 focus on affordable and equitable access for all.	Bottom 2.5th-percentile prformer[96].
		090101Alt. Public infrastructure investment (% of GDP)	Ec	1.97	0.71	Target sets as 10% improvement on baseline value.	Finance Yearbook of China[64].
	9.2 Promote inclusive and sustainable industrialization and, by 2030, significantly raise industry's share of employment and gross domestic product, in line with national circumstances, and double its share in least developed countries	090201a. Industry production (% of GDP)	Ec	40.3	6.479	SDG Target 9.2 is to increase share. Average of 5 best performers[97,98].	Bottom 2.5th-percentile performer[97,98].
		090201b. Industry production per capita (Yuan/person)	Ec	179180	8300	SDG Target 9.2 is to increase share. Average of 5 best performers[97,98].	Bottom 2.5th-percentile performer[97,98].

		090202. Industry employment as share of total employment (%)	Ec	46.8	5.79	Target sets as Xu et al.[89].	Bottom 2.5th-percentile prformer[97].
	9.4 By 2030, upgrade infrastructure and retrofit industries to make them sustainable, with increased resource-use efficiency and greater adoption of clean and environmentally sound technologies and industrial processes, with all countries taking action in accordance with their respective capabilities	090401. CO2 emissions per unit of value added (kg/$ 2011)	En	0	0.9966	Target sets as Xu et al.[89].	Bottom 2.5th-percentile prformer[99].
SDG 10. Reduced Inequalities	10.1 By 2030, progressively achieve and sustain income growth of the bottom 40 per cent of the population at a rate higher than the national average	100101. Bottom 40% income growth to average income growth gap (average - bottom 40%)	Ec	0	8.185	SDG Target 10.1 is to achieve and sustain growth. Target sets as equal to income growth of national average.	Baseline projection.
		100101Alt1. Gini coefficient income (best 0–1 worst)	Ec	0.275	0.63	SDG Index and Dashboards Report[80].	SDG Index and Dashboards Report[80].
		100101Alt2. Palma ratio income (top 10%/bottom 40%)	Ec	0.9	2.5	SDG Index and Dashboards Report[80].	SDG Index and Dashboards Report[80].
	10.2 By 2030, empower and promote the social, economic and political inclusion of all, irrespective of age, sex, disability, race, ethnicity, origin, religion or economic or other status	100201. Proportion of population below half median income (%)	Ec	5	24.5	Average of 5 best performers[100].	Bottom 2.5th-percentile pformer[100].
	10.4 Adopt policies, especially fiscal, wage and social protection policies, and progressively achieve greater equality	100401. Average labor share (worst 0–1 best)	Ec	0.674	0.275	Average of 5 best performers[101].	Bottom 2.5th-percentile prformer[101].
SDG 11. Sustainable Cities and Communties	11.5 By 2030, significantly reduce the number of deaths and the number of people affected and substantially decrease the direct economic losses relative to global gross domestic product caused by disasters, including water-related disasters, with a focus on protecting the poor and people in vulnerable situations	110501a. Mortality due to disasters five year (per 100,000 population, 5-year average)	S	0.2745	1.476	Sendai Framework DRR targets for 2030 are to "substantially reduce"[81]; target sets as 50% improvement on baseline value.	China Statistical Yearbook[15]; China Statistical Yearbook on Environment[82].
		110501b. Proportion of population affected by natural disasters (%,	S	5.658	32.08	Sendai Framework DRR targets for 2030 are to	China Statistical Yearbook[15]; China

		5-year average)				"substantially reduce"[81]; target sets as 50% improvement on baseline value.	Statistical Yearbook on Environment[82].
		110502. Economic damage due to natural disasters (% of GDP, 5-year average)	Ec	0.39635	1.577	Sendai Framework DRR targets for 2030 are to "substantially reduce"[81]; target sets as 50% improvement on baseline value.	China Statistical Yearbook[15]; China Statistical Yearbook on Environment[82].
	11.6 By 2030, reduce the adverse per capita environmental impact of cities, including by paying special attention to air quality and municipal and other waste management	110601. Proportion of urban waste collected and disposed (%)	En	100	50.78	SDG Target 11.6 already achieved for China.	China Statistical Yearbook[15].
		110602. PM 25 mean annual exposure (mcg/m^3)	En	6.3	87	SDG Index and Dashboards Report[80].	SDG Index and Dashboards Report[80].
SDG 12. Responsible Consumption and Production	12.2 By 2030, achieve the sustainable management and efficient use of natural resources	120201a. Material footprint per capita (t/person)	En	0.7809	109.07	Average of 5 best performers[94].	The lower bound is set based on an annual increase of 5.3% (estimated based on Global Material Flows Database[94] from 2000 to 2019) of material footprint per capita.
		120201b. Material footprint per unit of output (kg/$ 2011)	En	0.167	6.3252	Average of 5 performers[94].	Bottom 2.5th-percentile performer[94].
		120202a. Domestic material consumption per capita (t/person)	En	1.9	94.81	Average of 5 best performers[78].	The lower bound is set based on an annual increase of 4.7% (estimated based on Global Material Flows Database[94] from 2000 to 2019) of domestic material consumption per capita.
		120202b. Domestic material consumption per unit of output (kg/$ 2011)	En	0.181	3.845	Average of 5 best performers[95].	Bottom 2.5th-percentile performer[95].

	12.5 By 2030, substantially reduce waste generation through prevention, reduction, recycling and reuse	120501New. Average per capita waste generation (kg/person)	En	0.1	3.7	SDG Index and Dashboards Report[80].	SDG Index and Dashboards Report[80].
SDG 13. Climate Action	13.1 Strengthen resilience and adaptive capacity to climate-related hazards and natural disasters in all countries	130102a. Mortality due to disasters five year (per 100,000 population, 5-year average)	S	0.2745	1.476	Sendai Framework DRR targets for 2030 are to "substantially reduce"[81]; target sets as 50% improvement on baseline value.	China Statistical Yearbook[15]; China Statistical Yearbook on Environment[82].
		130102b. Proportion of population affected by natural disasters (%, 5-year average)	S	5.658	32.08	Sendai Framework DRR targets for 2030 are to "substantially reduce"[81]; target sets as 50% improvement on baseline value.	China Statistical Yearbook[15]; China Statistical Yearbook on Environment[82].
	13.2 Integrate climate change measures into national policies, strategies and planning	130201New1. Total greenhouse gas emissions (Gt)	En	10.46	17.5	Target sets as China's Long-Term Low-Carbon Development Strategies and Pathways[70].	Target set as Duan et al.[102].
		130201New2. Greenhouse gas emissions per capita (t/Year)	En	0.0485	22.9822	Average of 5 best performers[103].	Bottom 2.5th-percentile prformer[103].
		130201New3. CO2 emissions per unit value added (kg/$ 2011)	En	0	0.9966	Target sets as Xu et al.[89].	Bottom 2.5th-percentile prformer[99].
SDG 14. Life Below Water	14.4 By 2020, effectively regulate harvesting and end overfishing, illegal, unreported and unregulated fishing and destructive fishing practices and implement science-based management plans, in order to restore fish stocks in the shortest time feasible, at least to levels that can produce maximum sustainable yield as determined by their biological characteristics	140401. Proportion of fish stocks sustainably exploited (%)	En	74.81	0	Target sets as 50% improvement on baseline value.	Bottom 2.5th-percentile performer.
	14.5 By 2020, conserve at least 10 percent of coastal and marine areas, consistent with national	140501. Proportion of territorial waters effectively protected (%)	En	14	0	Target sets as 200% improvement on baseline	Bottom 2.5th-percentile performer.

	and international law and based on the best available scientific information					value.	
SDG 15. Life on Land	15.1 By 2020, ensure the conservation, restoration and sustainable use of terrestrial and inland freshwater ecosystems and their services, in particular forests, wetlands, mountains and drylands, in line with obligations under international agreements	150101. Forest cover (%)	En	63	0	Target sets as Xu et al.[89].	Bottom 2.5th-percentile performer[104].
		150102. Proportion of terrestrial areas effectively protected (%)	En	51.4	2.18	Average of 5 best performers[105].	Bottom 2.5th-percentile performer[105].
	15.5 Take urgent and significant action to reduce the degradation of natural habitats, halt the loss of biodiversity and, by 2020, protect and prevent the extinction of threatened species	150501. Red list index (worst 0–1 best)	En	1	0.6	SDG Index and Dashboards Report[80].	SDG Index and Dashboards Report[80].
SDG 16. Peace, Justice and Strong Institutions	16.1 Significantly reduce all forms of violence and related death rates everywhere	160101. Total mortality rates by cause -violence (per 1,000 population)	S	0.0016	0.106	SDG Target 16.1 is to "significantly reduce". Average of 3 best performers[83].	Bottom 2.5th-percentile performer[83].
	16.5 Substantially reduce corruption and bribery in all their forms	160502. Bribery incidence (best 0-1 worst)	S	0.0167	0.65	SDG Target 16.5 is to substantially reduce. Average of 3 best performers[106].	Bottom 2.5th-percentile performer[106].
	16.6 Develop effective, accountable and transparent institutions at all levels	160602. Normalized governance index (worst 0-1 best)	S	0.849	0.109	Average of 5 best performers[77].	Bottom 2.5th-percentile performer[77].
		160602Alt1. Government effectiveness (worst -2.5 to +2.5 best)	S	1.866	-1.922	Average of 5 best performers[77].	Bottom 2.5th-percentile performer[77].
		160602Alt2. Political stability (worst -2.5 to +2.5 best)	S	1.677	-2.5	Average of 5 best performers[77].	Bottom 2.5th-percentile performer[77].
SDG 17. Partnerships for the goals	17.1 Strengthen domestic resource mobilization, including through international support to developing countries, to improve domestic capacity for tax and other revenue collection	170101a. Domestic revenue (% of GDP)	Ec	40	10	SDG Index and Dashboards Report[80].	SDG Index and Dashboards Report[80].
		170102Alt.Government surplus or deficit (% of GDP)	Ec	4.78	-7.14	Average of 3 best performers[107].	Bottom 2.5th-percentile performers[107].
	17.4 Assist developing countries in attaining long-term debt sustainability through coordinated	170401. Interest on public debt (% of exports)	Ec	0.1272	27.81	Average of 5 best performers[108,109].	Bottom 2.5th-percentile performer[108,109].

	policies aimed at fostering debt financing, debt relief and debt restructuring, as appropriate, and address the external debt of highly indebted poor countries to reduce debt distress						

#Dimension for each indicator. Ec = economic; En = environmental; S = social.

Supplementary Table 5. Goodness-of-fit metrics of the simulated results for a selection of variables.

Variables	R2	MAE	MSE	RMSE	MAPE
Private saving	0.944	1.38E+12	2.97E+24	1.72E+12	0.097
Nominal GDP	0.997	1.25E+12	2.39E+24	1.55E+12	0.030
Total motor fuel consumption	0.707	3.21E+04	1.33E+09	3.64E+04	0.168
Total population	0.978	4.48E+06	4.26E+13	6.53E+06	0.003
Total industry production	0.990	7.36E+11	8.41E+23	9.17E+11	0.039
Total average years of schooling	0.934	0.115	0.021	0.145	0.014
Total final energy consumption	0.567	2.47E+05	8.11E+10	2.85E+05	0.151
Total exports	0.822	1.75E+12	4.78E+24	2.19E+12	0.156
Private consumption	0.982	1.03E+12	1.33E+24	1.15E+12	0.080
Total agriculture production	0.993	7.19E+10	8.04E+21	8.97E+10	0.015
GHG emissions	0.865	7.56E+08	7.72E+17	8.79E+08	0.101
Material footprint per catipa	0.946	0.895	1.134	1.065	0.060
Average life expectancy	0.821	0.637	0.510	0.714	0.009
Total electricity generation	0.972	300	1.06E+05	326	0.076
Domestic material consumption	0.712	3.93E+09	1.76E+19	4.20E+09	0.169
Proportion of population below national poverty line	0.981	0.014	0.000	0.019	0.276
Total services production	0.996	7.00E+11	6.08E+23	7.79E+11	0.030
Investment	0.965	1.85E+12	5.67E+24	2.38E+12	0.089

Supplementary Table 6. The long-term GDP projection of the iSDG-China model and the comparison against the GDP projections of several major models.

Models	Units	GDP Projections in 2050	Average annual GDP growth rate (%), 2020-2050
iSDG-China	Trillion 2015 US$	20.7–57.8	1.1–4.6
Jing et al.[62]	Trillion 2010 RMB	180–278	2.6–4.2
Jiang et al.[110]	Trillion 2015 US$	26.0–58.8	1.9–4.7
IIASA	Trillion 2005 US$ PPP	39.2–62.8	1.7–3.2
OECD	Trillion 2005 US$ PPP	39.1–86.2	2.1–4.8
PIK	Trillion 2005 US$ PPP	42.7–64.4	2.3–3.6

Supplementary Table 7. The average simulated results of SDGs under all states of the world by 2030 and 2050.

SDGs	SDG Indicators	Variables	2022	2030							2050						
				Baseline	Pandemic disease	Ageing and shrinking population	Deglobalization	Climate change	Biodiversity loss	Compound effects	Baseline	Pandemic disease	Ageing and shrinking population	Deglobalization	Climate change	Biodiversity loss	Compound effects
1	010101	Proportion of population below poverty line (%)	0.005	0.003	0.003	0.003	0.003	0.003	0.003	0.003	0.001	0.001	0.001	0.001	0.001	0.001	0.002
	010201	Proportion of population below national poverty line (%)	0.008	0.004	0.004	0.004	0.004	0.004	0.004	0.004	0.001	0.001	0.002	0.002	0.001	0.001	0.002
	010401	Average access to basic health care (worst 0-1 best)	0.805	0.857	0.855	0.829	0.818	0.858	0.858	0.789	0.897	0.896	0.736	0.797	0.903	0.898	0.652
	010501a	Mortality due to disasters five year (per 100,000 population, 5-year average)	0.560	0.614	0.614	0.614	0.614	0.622	0.614	0.622	0.723	0.723	0.723	0.723	0.767	0.723	0.767
	010501b	Proportion of population affected by natural disasters (%, 5-year average)	11.5	12.7	12.7	12.7	12.7	12.8	12.7	12.8	14.9	14.9	14.9	14.9	15.8	14.9	15.8
	010502	Economic damage due to natural disasters (% of GDP, 5-year average)	0.808	0.887	0.887	0.887	0.887	0.899	0.887	0.899	1.045	1.045	1.045	1.045	1.108	1.045	1.108
2	020101	Prevalence of undernourishment (%)	0.003	0.002	0.002	0.002	0.002	0.002	0.002	0.002	0.001	0.001	0.001	0.001	0.000	0.001	0.001
	020201	Prevalence of stunting (%)	0.077	0.076	0.076	0.076	0.076	0.076	0.076	0.076	0.074	0.074	0.075	0.075	0.074	0.074	0.075
	020202	Prevalence of malnutrition (%)	0.017	0.016	0.016	0.016	0.016	0.016	0.016	0.016	0.016	0.016	0.016	0.016	0.016	0.016	0.016
	020301	Total agriculture production per labor unit (t/person)	10.4	14.0	14.0	13.9	13.7	14.0	14.0	13.6	16.4	16.4	17.3	17.5	16.6	16.4	17.7
	020301ALT	Cereal yield (t/ha)	5.662	6.036	6.033	5.983	5.944	6.045	6.036	5.899	5.923	5.920	5.507	5.740	6.044	5.921	5.516
	020401	Proportion of harvested area sustainably managed (% of harvested area)	2.658	3.327	3.327	3.327	3.327	3.327	3.327	3.327	5.000	5.000	5.000	5.000	5.000	5.000	5.000
3	030101	Maternal mortality ratio (per 100,000 population)	44.5	45.7	45.7	64.7	45.9	45.7	45.7	65.0	39.2	39.2	56.9	39.7	39.2	39.2	57.4
	030102	Average access to basic health care (worst 0-1 best)	0.805	0.857	0.855	0.829	0.818	0.858	0.858	0.789	0.897	0.896	0.736	0.797	0.903	0.898	0.652
	030201	Under five mortality rate (per 1,000 population)	16.8	15.3	15.3	15.3	15.5	15.3	15.3	15.5	13.4	13.4	14.1	13.8	13.4	13.4	14.6
	030202	Neonatal mortality rate (per 1,000 population)	7.211	6.419	6.421	6.466	6.538	6.418	6.418	6.583	5.359	5.361	5.792	5.605	5.340	5.357	6.013
	030301	Annual deaths due to COVID-19 (per 100,000 population)	0.043	0	10.041	0	0	0	0	9.028	0	10.547	0	0	0	0	10.093
	030401	Cardiovascular neoplasm diabetes and respiratory mortality (per 1,000 population)	3.775	4.261	4.253	4.407	4.379	4.261	4.259	4.519	5.969	5.948	6.955	6.274	5.947	5.967	7.240
	030601	Total mortality rates by cause -road (per 1,000 population)	0.134	0.125	0.126	0.130	0.131	0.125	0.125	0.136	0.167	0.167	0.186	0.173	0.166	0.167	0.195
	030701	Contraceptive prevalence rate (%)	90.7	92.1	92.1	92.0	91.8	92.1	92.1	91.7	95.7	95.7	94.3	94.6	95.8	95.7	93.6
	030702	Adolescent birth rate (per 1,000 population)	10.5	9.2	9.2	6.6	9.2	9.2	9.2	6.6	7.5	7.5	5.4	7.5	7.5	7.5	5.4
	030801	Average access to basic health care (worst 0-1 best)	0.805	0.857	0.855	0.829	0.818	0.858	0.858	0.789	0.897	0.896	0.736	0.797	0.903	0.898	0.652
	030801ALT	Life expectancy at birth (year)	78.2	79.4	79.2	79.3	79.2	79.4	79.4	78.9	81.1	81.0	80.5	80.7	81.1	81.1	80.0

4	040101	Proportion of population completed secondary school (% of population age 20 to 24)	83.8	89.9	89.9	89.9	89.9	89.9	89.9	89.9	90.8	90.8	86.9	90.8	90.8	90.8	86.9
	040301	Proportion of population enrolled in tertiary education (% of population age 20 to 29)	17.6	29.7	29.7	29.7	29.6	29.7	29.7	29.5	39.9	39.8	35.5	35.5	40.1	40.0	32.5
	040301ALT	Women proportion of total tertiary graduates (%)	41.2	43.0	43.0	43.0	42.9	43.0	43.0	42.9	47.2	47.2	46.7	47.1	47.2	47.2	46.6
	040501	Adult literacy gender gap ratio (best 0-1 worst)	0.032	0.024	0.024	0.024	0.024	0.024	0.024	0.024	0.011	0.011	0.012	0.011	0.011	0.011	0.012
	040601	Average adult literacy rate (% of total population)	92.2	93.9	93.9	93.9	93.9	93.9	93.9	93.9	96.8	96.8	96.7	96.9	96.8	96.8	96.8
	040601ALT	Total average years of schooling (year)	9.8	10.5	10.5	10.5	10.5	10.5	10.5	10.5	12.2	12.2	12.0	12.1	12.2	12.2	11.9
5	050501	Female share of employment in managerial positions (%)	31.1	33.9	33.9	33.9	33.9	33.9	33.9	33.9	38.9	38.9	38.7	38.8	38.9	38.9	38.6
	050501ALT	Employment to population ratio (female/male parity)	0.860	0.866	0.866	0.866	0.866	0.866	0.866	0.866	0.875	0.875	0.875	0.875	0.875	0.875	0.875
	050601	Contraceptive prevalence rate (%)	90.7	92.1	92.1	92.0	91.8	92.1	92.1	91.7	95.7	95.7	94.3	94.6	95.8	95.7	93.6
6	060101	Average access to safely managed water source (worst 0-1 best)	0.888	0.887	0.886	0.899	0.872	0.880	0.887	0.876	0.996	0.996	0.978	0.941	0.952	0.996	0.854
	060201	Average access to safely managed sanitation facility (worst 0-1 best)	0.736	0.904	0.902	0.903	0.864	0.905	0.905	0.863	1.000	1.000	0.995	0.980	1.000	1.000	0.912
	060402	Water resources vulnerability index (best 0-1 worst)	0.239	0.260	0.260	0.250	0.247	0.274	0.260	0.249	0.272	0.272	0.230	0.248	0.336	0.272	0.245
7	070101	Population with access to electricity (%)	100	100	100	100	100	100	100	100	100	100	100	100	100	100	100
	070201	Share of renewable energy in total primary energy supply (%)	9.7	16.9	16.9	16.1	16.3	16.9	16.9	15.3	20.9	20.9	20.3	20.5	21.1	20.9	19.9
	070201ALT	Renewable share in electricity (%)	32.1	51.4	51.4	50.8	48.8	51.4	51.4	47.5	59.1	59.1	57.2	58.0	59.4	59.1	56.5
	070202	CO2 emissions from fuel combustion for electricity per total electricity output (Mt/TWh)	0.473	0.215	0.216	0.224	0.249	0.220	0.215	0.271	0.090	0.090	0.108	0.100	0.099	0.090	0.133
	070301	Energy intensity level of primary energy (Mj/$ 2011)	5.147	4.744	4.741	4.776	4.662	4.812	4.744	4.769	5.096	5.090	4.590	4.665	5.300	5.100	4.514
8	080101	Real GDP growth rate per capita (%)	4.769	5.555	5.548	4.144	4.570	5.591	5.568	3.317	4.075	4.069	2.680	3.299	4.300	4.077	2.295
	080101ALT1	Real GDP per capita (2015 Yuan/person)	7.02E+04	1.16E+05	1.15E+05	1.07E+05	1.02E+05	1.16E+05	1.16E+05	9.45E+04	2.85E+05	2.84E+05	1.88E+05	2.12E+05	2.93E+05	2.85E+05	1.49E+05
	080201	Real GDP per employed person (2015 Yuan/person)	1.32E+05	2.12E+05	2.12E+05	1.94E+05	1.90E+05	2.12E+05	2.12E+05	1.74E+05	4.85E+05	4.84E+05	3.43E+05	3.93E+05	4.95E+05	4.86E+05	2.88E+05
	080401b	Material footprint per unit of output (kg/$ 2011)	1.415	1.205	1.205	1.232	1.248	1.207	1.204	1.278	0.955	0.955	1.052	1.022	0.953	0.954	1.119
	080402b	Domestic material consumption per unit of output (kg/$ 2011)	1.496	1.296	1.297	1.318	1.319	1.324	1.299	1.372	0.887	0.888	1.052	0.990	0.947	0.888	1.198
	080502	Unemployment rate (%)	7.355	7.187	7.208	8.256	8.837	7.180	7.185	9.761	2.425	2.469	12.291	10.361	1.732	2.398	17.034
	080502ALT	Average employment to adult population ratio (%)	64.4	64.5	64.5	63.8	63.4	64.5	64.5	62.7	67.8	67.8	61.0	62.3	68.3	67.9	57.7
	080601	Youth not in education employment or training (%)	12.5	6.453	6.467	6.453	6.453	6.453	6.453	6.496	1.198	1.200	2.871	1.714	1.198	1.198	9.859

	090101	Rural access index (worst 0-1 best)	0.830	0.988	0.987	0.983	0.977	0.987	0.988	0.972	1.000	1.000	1.000	1.000	1.000	1.000	1.000
	090101ALT	Public infrastructure investment (% of GDP)	1.169	1.193	1.189	1.193	1.193	1.193	1.193	1.188	1.193	1.192	1.193	1.193	1.193	1.193	1.191
	090201a	Industry production (% of GDP)	39.5	39.2	39.2	39.1	38.9	39.2	39.2	38.7	37.7	37.7	38.4	37.6	37.7	37.7	38.2
9	090201b	Industry production per capita (Yuan/person)	2.77E+04	4.54E+04	4.53E+04	4.18E+04	3.96E+04	4.54E+04	4.54E+04	3.66E+04	1.07E+05	1.07E+05	7.22E+04	7.98E+04	1.10E+05	1.08E+05	5.69E+04
	090202	Industry employment as share of total employment (%)	28.1	26.2	26.1	26.5	26.7	26.2	26.2	27.0	16.8	16.8	19.9	18.9	16.6	16.8	21.3
	090401	CO2 emissions per unit of value added (kg/$ 2011)	0.403	0.274	0.274	0.281	0.282	0.284	0.274	0.302	0.160	0.160	0.159	0.156	0.186	0.160	0.187
	100101	Bottom 40% income growth to average income growth gap (average - bottom 40%)	0.014	-0.002	-0.002	-0.002	-0.002	-0.002	-0.002	-0.002	0.006	0.006	0.004	0.005	0.006	0.006	0.004
	100101ALT1	Gini coefficient income (best 0-1 worst)	0.371	0.362	0.362	0.362	0.361	0.362	0.362	0.361	0.373	0.373	0.370	0.371	0.373	0.372	0.370
10	100101ALT2	Palma ratio income (top 10%/bottom 40%)	1.419	1.366	1.366	1.363	1.358	1.366	1.365	1.356	1.446	1.446	1.427	1.432	1.448	1.445	1.425
	100201	Proportion of population below half median income (%)	0.900	0.891	0.890	0.888	0.888	0.891	0.891	0.886	0.729	0.729	0.705	0.712	0.730	0.729	0.695
	100401	Average labor share (worst 0-1 best)	0.373	0.368	0.368	0.370	0.370	0.368	0.368	0.373	0.354	0.354	0.364	0.360	0.354	0.354	0.367
	110501a	Mortality due to disasters five year (per 100,000 population, 5-year average)	0.560	0.614	0.614	0.614	0.614	0.622	0.614	0.622	0.723	0.723	0.723	0.723	0.767	0.723	0.767
	110501b	Proportion of population affected by natural disasters (%, 5-year average)	11.538	12.662	12.662	12.662	12.662	12.831	12.662	12.831	14.915	14.915	14.915	14.915	15.813	14.915	15.813
11	110502	Economic damage due to natural disasters (% of GDP, 5-year average)	0.808	0.887	0.887	0.887	0.887	0.899	0.887	0.899	1.045	1.045	1.045	1.045	1.108	1.045	1.108
	110601	Proportion of urban waste collected and disposed (%)	91.9	100.0	100.0	100.0	100.0	100.0	100.0	100.0	100.0	100.0	100.0	100.0	100.0	100.0	100.0
	110602	PM 25 mean annual exposure (mcg/m^3)	42.2	41.9	41.8	39.5	38.8	42.7	41.9	37.7	42.8	42.6	28.0	32.7	45.5	42.9	24.4
	120201a	Material footprint per capita (t/person)	24.0	33.7	33.7	31.8	30.7	33.8	33.7	29.2	65.7	65.6	47.8	52.3	67.5	65.8	40.3
	120201b	Material footprint per unit of output (kg/$ 2011)	1.415	1.205	1.205	1.232	1.248	1.207	1.204	1.278	0.955	0.955	1.052	1.022	0.953	0.954	1.119
12	120202a	Domestic material consumption per capita (t/person)	25.4	36.3	36.2	34.0	32.5	37.1	36.4	31.4	61.1	61.0	47.8	50.6	67.0	61.3	43.1
	120202b	Domestic material consumption per unit of output (kg/$ 2011)	1.496	1.296	1.297	1.318	1.319	1.324	1.299	1.372	0.887	0.888	1.052	0.990	0.947	0.888	1.198
	120501	Average per capita waste generation (kg/person)	0.683	0.692	0.693	0.692	0.692	0.692	0.692	0.693	0.706	0.707	0.706	0.706	0.706	0.706	0.707
	130102a	Mortality due to disasters five year (per 100,000 population, 5-year average)	0.560	0.614	0.614	0.614	0.614	0.622	0.614	0.622	0.723	0.723	0.723	0.723	0.767	0.723	0.767
	130102b	Proportion of population affected by natural disasters (%, 5-year average)	11.5	12.7	12.7	12.7	12.7	12.8	12.7	12.8	14.9	14.9	14.9	14.9	15.8	14.9	15.8
13	130201	Total greenhouse gas emissions (Gt)	10.0	11.4	11.3	10.6	10.3	11.8	11.4	10.0	15.7	15.6	9.4	11.4	18.7	15.7	8.8
	130201ALT1	Greenhouse gas emissions per capita (t/Year)	6.843	7.656	7.644	7.263	6.938	7.947	7.664	6.908	10.999	10.962	7.231	8.019	13.148	11.027	6.764
	130201ALT2	CO2 emissions per unit value added (kg/$ 2011)	0.403	0.274	0.274	0.281	0.282	0.284	0.274	0.302	0.160	0.160	0.159	0.156	0.186	0.160	0.187

14	140401	Proportion of fish stocks sustainably exploited (%)	46.9	42.4	42.5	42.5	42.6	42.4	42.4	42.6	40.2	40.2	41.3	41.0	40.2	40.2	42.0
	140501	Proportion of territorial waters effectively protected (%)	3.783	4.484	4.459	4.117	3.983	4.489	4.242	3.453	6.065	6.033	3.737	4.538	6.223	4.625	2.250
15	150101	Forest cover (%)	20.4	20.8	20.7	20.7	20.6	20.7	20.2	20.1	23.4	23.2	21.3	21.6	23.3	21.2	18.8
	150102	Proportion of terrestrial areas effectively protected (%)	35.7	36.9	36.3	34.9	33.9	36.5	29.1	26.0	39.8	39.1	27.2	31.6	40.0	29.5	17.0
	150501	Red list index (worst 0-1 best)	0.730	0.714	0.714	0.713	0.713	0.705	0.711	0.703	0.685	0.684	0.676	0.677	0.656	0.676	0.636
16	160101	Total mortality rates by cause -violence (per 1,000 population)	0.030	0.030	0.030	0.030	0.030	0.030	0.030	0.030	0.030	0.030	0.030	0.030	0.030	0.030	0.031
	160502	Bribery incidence (best 0-1 worst)	0.058	0.058	0.058	0.058	0.058	0.058	0.058	0.058	0.058	0.058	0.058	0.058	0.058	0.058	0.058
	160602	Normalized governance index (worst 0-1 best)	0.439	0.439	0.439	0.439	0.439	0.439	0.439	0.439	0.439	0.439	0.439	0.439	0.439	0.439	0.439
	160602ALT1	Government effectiveness (worst -2.5 to +2.5 best)	0.595	0.595	0.595	0.595	0.595	0.595	0.595	0.595	0.595	0.595	0.595	0.595	0.595	0.595	0.595
	160602ALT2	Political stability (worst -2.5 to +2.5 best)	0.448	0.448	0.448	0.448	0.448	0.4	0.448	0.448	0.448	0.448	0.448	0.448	0.448	0.448	0.448
17	170101a	Domestic revenue (% of GDP)	19.7	20.7	20.7	20.6	20.8	20.7	20.7	20.7	23.1	23.1	23.0	23.1	23.1	23.1	23.0
	170102ALT	Government surplus or deficit (% of GDP)	-4.277	-3.272	-3.264	-3.365	-3.464	-3.267	-3.212	-3.518	-0.543	-0.540	-0.898	-0.861	-0.521	-0.471	-1.265
	170401	Interest on public debt (% of export)	3.837	3.612	3.617	4.239	5.280	3.603	3.586	6.097	2.795	2.800	5.830	5.557	2.665	2.725	10.313

References

1. Bogoch, I. I. *et al.* Potential for global spread of a novel coronavirus from China. *J. Travel Med.* **27**, taaa011 (2020).
2. Roberton, T. *et al.* Early estimates of the indirect effects of the COVID-19 pandemic on maternal and child mortality in low-income and middle-income countries: a modelling study. *Lancet Glob. Health* **8**, e901–e908 (2020).
3. WHO. *WHO Coronavirus (COVID-19) Dashboard.* https://covid19.who.int (World Health Organization, 2022).
4. IMF. *Fiscal Monitor: Policies for the Recovery*. (International Monetary Fund, 2020).
5. FAO, IFAD, UNICEF & WHO. *The State of Food Security and Nutrition in the World 2020. Transforming Food Systems for Affordable Healthy Diets*. (FAO, 2020).
6. National Bureau of Statistics. The Urban Surveyed Unemployment Rate. https://data.stats.gov.cn/easyquery.htm?cn=A01 (2022).
7. Berg, J., Hilal, A., El, S. & Horne, R. *World Employment and Social Outlook: Trends 2021*. (International Labour Organization, 2021).
8. Telenti, A. *et al.* After the pandemic: perspectives on the future trajectory of COVID-19. *Nature* **596**, 495–504 (2021).
9. WHO. Tracking SARS-CoV-2 variants. https://www.who.int/activities/tracking-SARS-CoV-2-variants (World Health Organization, 2020).
10. Lopez Bernal, J. *et al.* Effectiveness of Covid-19 Vaccines against the B.1.617.2 (Delta) Variant. *N. Engl. J. Med.* **385**, 585–594 (2021).
11. Baker, R. E. *et al.* Infectious disease in an era of global change. *Nat. Rev. Microbiol.* **20**, 193–205 (2022).
12. Demeny, P. G. & McNicoll, G. *Encyclopedia of Population*. (Macmillan Reference USA, 2003).
13. Hu, Z., Peng, X. & Wu, Y. On the “Chinese Approach” to Actively Addressing Population Ageing. *Soc. Sci. China* 46-66+205 (2022).
14. UNPD. *World Urbanization Prospects: The 2018 Revision*. (United Nations Department of Economic and Social Affairs Population Division, 2018).
15. National Bureau of Statistics. *China Statistical Yearbook*. (China Statistics Press, 2021).
16. Jarzebski, M. P. *et al.* Ageing and population shrinking: implications for sustainability in the urban century. *Npj Urban Sustain.* **1**, 1–11 (2021).
17. Ye, Y. When will China’s population peak? It depends who you ask. *Nature* (2022) doi:10.1038/d41586-022-02304-8.
18. Bloom, D. E. *et al.* Macroeconomic implications of population ageing and selected policy responses. *Lancet Lond. Engl.* **385**, 649–657 (2015).
19. Dixon, S. Implications of population ageing for the labour market. *Labour Mark. Trends* **111**, 67–76 (2003).
20. D’Addio, A. C., Keese, M. & Whitehouse, E. Population ageing and labour markets. *Oxf. Rev. Econ. Policy* **26**, 613–635 (2010).
21. Sharpe, A. Is ageing a drag on productivity growth? A review article on ageing, health and productivity: The economics of increased life expectancy. *Int. Product. Monit.* 82 (2011).

22. WHO. *Prevention and Control of Outbreaks of Seasonal Influenza in Long-Term Care Facilities: A Review of the Evidence and Best-Practice Guidance*. (World health organization Regional Office for Europe, 2017).
23. Hank, K. Societal determinants of productive aging: a multilevel analysis across 11 European countries. *Eur. Sociol. Rev.* **27**, 526–541 (2011).
24. Cristea, M. & Mitrică, A. Global ageing: Do privately managed pension funds represent a long term alternative for the Romanian pension system? Empirical research. *Romanian J. Polit. Sci.* **16**, 63–106 (2016).
25. Wikipedia. *Deglobalization*. https://en.wikipedia.org/wiki/Deglobalization (Wikipedia, 2023).
26. Khor, M. *Globalization and the South: Some Critical Issues*. (Third World Network Penang, 2000).
27. Rodrik, D. Populism and the economics of globalization. *J. Int. Bus. Policy* **1**, 12–33 (2018).
28. UNCTAD. *Global Investment Trends Monitor 2021*. (United Nations Conference on Trade and Development, 2021).
29. Deb, P., Furceri, D., Ostry, J. D. & Tawk, N. The economic effects of COVID-19 containment measures. *Open Econ. Rev.* **33**, 1–32 (2022).
30. Prohorovs, A. Russia's war in Ukraine: Consequences for European countries' businesses and economies. *J. Risk Financ. Manag.* **15**, 295 (2022).
31. Lardy, N. R. *The State Strikes Back: The End of Economic Reform in China?* (Peterson Institute for International Economics, 2019).
32. Center for Macroeconomic Research at Xiamen University. *China's Macroeconomic Outlook: Quarterly Forecast and Analysis Report, March 2020*. (Springer, Singapore, 2020). doi:10.1007/978-981-15-9279-9.
33. Masson-Delmotte, V. *et al. Global Warming of 1.5 C: IPCC Special Report on Impacts of Global Warming of 1.5 C above Pre-Industrial Levels in Context of Strengthening Response to Climate Change, Sustainable Development, and Efforts to Eradicate Poverty*. (Cambridge University Press, 2022).
34. Trenberth, K. E. Changes in precipitation with climate change. *Clim. Res.* **47**, 123–138 (2011).
35. Annex, I. Managing the risks of extreme events and disasters to advance climate change adaptation. *Sciences* **10**, 97–104 (2012).
36. Milne, G. A., Gehrels, W. R., Hughes, C. W. & Tamisiea, M. E. Identifying the causes of sea-level change. *Nat. Geosci.* **2**, 471–478 (2009).
37. Gattuso, J.-P. *et al.* Contrasting futures for ocean and society from different anthropogenic CO2 emissions scenarios. *Science* **349**, aac4722 (2015).
38. Urban, M. C. Accelerating extinction risk from climate change. *Science* **348**, 571–573 (2015).
39. Olhoff, A. & Christensen, J. M. *Emissions Gap Report 2020*. (United Nations Environment Programme, 2020).
40. Cardinale, B. J. *et al.* Biodiversity loss and its impact on humanity. *Nature* **486**, 59–67 (2012).
41. Butchart, S. H. M. *et al.* Global Biodiversity: Indicators of Recent Declines. *Science* **328**, 1164–1168 (2010).
42. WRI. *Millennium Ecosystem Assessment: Ecosystems and Human Well-Being*. (World Resources Institute, 2005).

43. Bellard, C., Bertelsmeier, C., Leadley, P., Thuiller, W. & Courchamp, F. Impacts of climate change on the future of biodiversity. *Ecol. Lett.* **15**, 365–377 (2012).
44. Schipper, J. *et al.* The status of the world's land and marine mammals: diversity, threat, and knowledge. *Science* **322**, 225–230 (2008).
45. Fahrig, L. Effects of habitat fragmentation on biodiversity. *Annu. Rev. Ecol. Evol. Syst.* **34**, 487–515 (2003).
46. Liu, J. *et al.* Ecological degradation in protected areas: the case of Wolong Nature Reserve for giant pandas. *Science* **292**, 98–101 (2001).
47. Xu, W. *et al.* Strengthening protected areas for biodiversity and ecosystem services in China. *Proc. Natl. Acad. Sci.* **114**, 1601–1606 (2017).
48. Bryan, B. A. *et al.* China's response to a national land-system sustainability emergency. *Nature* **559**, 193–204 (2018).
49. Kermack, W. O., McKendrick, A. G. & Walker, G. T. A contribution to the mathematical theory of epidemics. *Proc. R. Soc. Lond. Ser. Contain. Pap. Math. Phys. Character* **115**, 700–721 (1927).
50. Lipsitch, M. *et al.* Transmission dynamics and control of severe acute respiratory syndrome. *Science* **300**, 1966–1970 (2003).
51. Wu, J. T., Leung, K. & Leung, G. M. Nowcasting and forecasting the potential domestic and international spread of the 2019-nCoV outbreak originating in Wuhan, China: a modelling study. *The Lancet* **395**, 689–697 (2020).
52. Zhao, J. *et al.* Disease Burden Attributable to the First Wave of COVID-19 in China and the Effect of Timing on the Cost-Effectiveness of Movement Restriction Policies. *Value Health* **24**, 615–624 (2021).
53. Tang, B. *et al.* Estimation of the Transmission Risk of the 2019-nCoV and Its Implication for Public Health Interventions. *J. Clin. Med.* **9**, 462 (2020).
54. Costris-Vas, C., Schwartz, E. J. & Smith, R. Predicting COVID-19 using past pandemics as a guide: how reliable were mathematical models then, and how reliable will they be now? *Math. Biosci. Eng. MBE* **17**, 7502–7518 (2020).
55. Cai, J. *et al.* Modeling transmission of SARS-CoV-2 Omicron in China. *Nat. Med.* **28**, 1468–1475 (2022).
56. Millennium Institute. *iSDG Documentation*. (Millennium Institute, 2021).
57. Moallemi, E. A. *et al.* Early systems change necessary for catalyzing long-term sustainability in a post-2030 agenda. *One Earth* **5**, 792–811 (2022).
58. CDC. Past Seasons Estimated Influenza Disease Burden. https://www.cdc.gov/flu/about/burden/past-seasons.html (Centers for Disease Control and Prevention, 2020).
59. Wei, C. Forecasting Negative Population Growth and Population Ageing in China. *Soc. Sci. J.* (2022).
60. WHO. UHC Service Coverage Index (SDG 3.8.1). https://www.who.int/data/gho/data/indicators/indicator-details/GHO/uhc-index-of-service-coverage. (World Health Organization, 2023).
61. Dieleman, J. L. *et al.* National spending on health by source for 184 countries between 2013 and 2040. *The Lancet* **387**, 2521–2535 (2016).
62. Jing, C. *et al.* Gridded value-added of primary, secondary and tertiary industries in China under Shard Socioeconomic Pathways. *Sci. Data* **9**, 309 (2022).

63. World Bank. World Development Indicators. https://datatopics.worldbank.org/world-development-indicators/ (World Bank, 2022).
64. Ministry of Finance of the People's Republic of China. *Finance Yearbook of China*. (China Financial & Economic Publishing House, 2020).
65. Ekins, P. *et al.* Resource efficiency: Potential and economic implications. (2016).
66. Giljum, S. & Polzin, C. Resource efficiency for sustainable growth: global trends and European policy scenarios. *Backgr. Pap. Sustain. Eur. Res. Inst. SERI Vienna* (2009).
67. Organisation for Economic Co-operation and Development Staff. *OECD Environmental Outlook to 2050: The Consequences of Inaction*. (OECD Publishing, 2012).
68. Yang, J. *et al.* Projecting heat-related excess mortality under climate change scenarios in China. *Nat. Commun.* **12**, 1039 (2021).
69. MOE. The Communist Party of China Central Committee and the State Council recen tly issued China's education modernization 2035. http://www.moe.gov.cn/jyb_xwfb/gzdt_gzdt/201902/t20190223_370857.html (Ministry of Education of the People's Republic of China, 2019).
70. Institute of Climate Change and Sustainable Development at Tsinghua University. *China's Long-Term Low-Carbon Development Strategies and Pathways: Comprehensive Report*. (Springer, 2022).
71. National Forestry and Grassland Administration. *China Forestry and Grassland Statistical Yearbook*. (Forestry Publishing House, 2019).
72 UNEP. COP15: Nations Adopt Four Goals, 23 Targets for 2030 In Landmark UN Biodiversity Agreement. https://www.cbd.int/article/cop15-cbd-press-release-final-19dec2022.(United Nations Environment Programme, 2022).
73. Lu, N. *et al.* Biophysical and economic constraints on China's natural climate solutions. *Nat. Clim. Change* **12**, 847–853 (2022).
74. O'Neill, B. C. *et al.* The roads ahead: Narratives for shared socioeconomic pathways describing world futures in the 21st century. *Glob. Environ. Change* **42**, 169–180 (2017).
75. United Nations. *World Population Prospects 2022: Summary of Results*. (United Nations, 2022).
76. Allen, C., Metternicht, G., Wiedmann, T. & Pedercini, M. Greater gains for Australia by tackling all SDGs but the last steps will be the most challenging. *Nat. Sustain.* **2**, 1041–1050 (2019).
77. Kaufman, D. & Kraay, A. Worldwide Governance Indicators. http://info.worldbank.org/governance/wgi/#home (World Bank, 2022).
78. OECD. *Material Consumption*. https://www.oecd-ilibrary.org/environment/material-consumption/indicator/english_84971620-en (Organisation for Economic Co-operation and Development, 2019).
79. FAO. FAOSTAT database. https://www.fao.org/faostat/en/#home. (Food and Agriculture Organization of the United Nations, 2022).
80. Sachs, J., Kroll, C., Lafortune, G., Fuller, G. & Woelm, F. *Sustainable Development Report 2022*. (Cambridge University Press, 2022).
81. UNDRR. *Sendai Framework for Disaster Risk Reduction 2015-2030*. (United Nations Office for Disaster Risk Reduction, 2015).
82. National Bureau of Statistics & State Environmental Protection Administration of the

People's Republic of China. *China Statistical Yearbook on Environment*. (China Statistics Press, 2020).
83. WHO. WHO Mortality Database - WHO. https://www.who.int/data/data-collection-tools/who-mortality-database. (World Health Organization, 2020).
84. World Life Expectancy. Road traffic accidents death rate by country. https://www.worldlifeexpectancy.com/cause-of-death/road-traffic-accidents/by-country/.
85. Our World in Data. Gender gap in primary, secondary and tertiary education. https://ourworldindata.org/grapher/gender-gap-education-levels (*Our World in Data, 2023*).
86. Ministry of Education of the People's Republic of China. *Educational Statistics Yearbook of China*. (People's Education Press, 2020).
87. United Nations Development Programme. *Human Development Indices and Indicators: 2018 Statistical Update*. (UNDP, 2018).
88. OECD. Employment: Share of female managers. https://stats.oecd.org/index.aspx?%20queryid=96330. (Organisation for Economic Co-operation and Development, 2021).
89. Xu, Z. *et al.* Assessing progress towards sustainable development over space and time. *Nature* **577**, 74–78 (2020).
90. Our World in Data. Share of electricity production from renewables. https://ourworldindata.org/grapher/share-electricity-renewables (Our World in Data, 2023)
91. World Bank. Energy intensity level of primary energy. https://data.worldbank.org/indicator/EG.EGY.PRIM.PP.KD (World Bank).
92. World Bank. GDP per capita. https://data.worldbank.org/indicator/NY.GDP.PCAP.KD (World Bank).
93. World Bank. GDP per person employed (constant 2017 PPP $). https://data.worldbank.org/indicator/SL.GDP.PCAP.EM.KD (World Bank).
94. UNDP & IRP. *Global Material Flows Database*. (United Nations Development Programme, 2018).
95. OECD. *Material Productivity*. https://www.oecd-ilibrary.org/environment/material-productivity/indicator/english_dae52b45-en (Organisation for Economic Co-operation and Development, 2019).
96. Azavea, ReCAP & TRL. Rural Access Index. https://rai.azavea.com/ (Azavea, 2019).
97. World Bank. Employment in industry. https://data.worldbank.org/indicator/SL.IND.EMPL.ZS (World Bank).
98. OECD. *Industrial Production*. https://www.oecd-ilibrary.org/industry-and-services/industrial-production/indicator/english_39121c55-en (Organisation for Economic Co-operation and Development, 2018).
99. World Bank. CO2 emissions. https://data.worldbank.org/indicator/EN.ATM.CO2E.KD.GD (World Bank).
100. World Bank. Proportion of people living below 50 percent of median income (%). https://data.worldbank.org/indicator/SI.DST.50MD (World Bank).
101. ILO. ILOSTAT database. https://ilostat.ilo.org/data/ (International Labour Organization).
102. Duan, H. *et al.* Assessing China's efforts to pursue the 1.5°C warming limit. *Science* **372**, 378–385 (2021).
103. Andrew, R. M. A comparison of estimates of global carbon dioxide emissions from fossil

carbon sources. *Earth Syst. Sci. Data* **12**, 1437–1465 (2020).

104. FAO. Forest area as a percentage of total land area. https://www.fao.org/sustainable-development-goals/indicators%20/1511/en/ (Food and Agriculture Organization of the United Nations).
105. World Bank. Terrestrial protected areas (% of total land area). https://data.worldbank.org/indicator/ER.LND.PTLD.ZS (World Bank).
106. Transparency International. Global Corruption Barometer Series 2017. https://www.transparency.org/en/gcb/global/global-corruption-barometer-2017 (Transparency International, 2017).
107. OECD. *General Government Deficit*. https://www.oecd-ilibrary.org/governance/general-government-deficit/indicator/english_77079edb-en (Organisation for Economic Co-operation and Development, 2018).
108. World Bank. Interest payments (current LCU). https://data.worldbank.org/indicator/GC.XPN.INTP.CN (World Bank).
109. World Bank. Exports of goods and services (current LCU). https://data.worldbank.org/indicator/NE.EXP.GNFS.CN (World Bank).
110. Jiang, D. *et al.* Future growth pattern projections under shared socioeconomic pathways: a municipal city bottom-up aggregated study based on a localised scenario and population projections for China. *Econ. Res.-Ekon. Istraživanja* **35**, 2574–2595 (2022).
