# Supplementary material for: Safeguarding China’s long-term sustainability against systemic disruptors
Source: Nat Commun. 2024 Jun 24;15:5338. doi: 10.1038/s41467-024-49725-9 (PMC11196269; doi:10.1038/s41467-024-49725-9)
Supplement: Supplementary file 3 — Reporting Summary [file 41467_2024_49725_MOESM3_ESM.pdf]

nature portfolio

Corresponding author(s): Yucheng Dong
Last updated by author(s): Jun 17, 2024

Reporting Summary

Nature Portfolio wishes to improve the reproducibility of the work that we publish. This form provides structure for consistency and transparency in reporting. For further information on Nature Portfolio policies, see our Editorial Policies and the Editorial Policy Checklist.

Statistics

For all statistical analyses, confirm that the following items are present in the figure legend, table legend, main text, or Methods section.

n/a	Confirmed	
☐	☒	The exact sample size (*n*) for each experimental group/condition, given as a discrete number and unit of measurement
☒	☐	A statement on whether measurements were taken from distinct samples or whether the same sample was measured repeatedly
☒	☐	The statistical test(s) used AND whether they are one- or two-sided<br>*Only common tests should be described solely by name; describe more complex techniques in the Methods section.*
☒	☐	A description of all covariates tested
☒	☐	A description of any assumptions or corrections, such as tests of normality and adjustment for multiple comparisons
☒	☐	A full description of the statistical parameters including central tendency (e.g. means) or other basic estimates (e.g. regression coefficient) AND variation (e.g. standard deviation) or associated estimates of uncertainty (e.g. confidence intervals)
☒	☐	For null hypothesis testing, the test statistic (e.g. *F*, *t*, *r*) with confidence intervals, effect sizes, degrees of freedom and *P* value noted<br>*Give P values as exact values whenever suitable.*
☒	☐	For Bayesian analysis, information on the choice of priors and Markov chain Monte Carlo settings
☒	☐	For hierarchical and complex designs, identification of the appropriate level for tests and full reporting of outcomes
☒	☐	Estimates of effect sizes (e.g. Cohen's *d*, Pearson's *r*), indicating how they were calculated

Our web collection on statistics for biologists contains articles on many of the points above.

Software and code

Policy information about availability of computer code

Data collection	No software was used for data collection.
Data analysis	The codes used to produce the results of the study are available in the public repository (https://doi.org/10.5281/zenodo.11897708).

For manuscripts utilizing custom algorithms or software that are central to the research but not yet described in published literature, software must be made available to editors and reviewers. We strongly encourage code deposition in a community repository (e.g. GitHub). See the Nature Portfolio guidelines for submitting code & software for further information.

Data

Policy information about availability of data

All manuscripts must include a data availability statement. This statement should provide the following information, where applicable:

- Accession codes, unique identifiers, or web links for publicly available datasets
- A description of any restrictions on data availability
- For clinical datasets or third party data, please ensure that the statement adheres to our policy

The dataset used for model calibration in the study is available in the public repository (https://doi.org/10.5281/zenodo.11443162). Additional materials and data are available in the Supplementary Information.

Research involving human participants, their data, or biological material

Policy information about studies with human participants or human data. See also policy information about sex, gender (identity/presentation), and sexual orientation and race, ethnicity and racism.

Reporting on sex and gender	N/A
Reporting on race, ethnicity, or other socially relevant groupings	N/A
Population characteristics	N/A
Recruitment	N/A
Ethics oversight	N/A

Note that full information on the approval of the study protocol must also be provided in the manuscript.

Field-specific reporting

Please select the one below that is the best fit for your research. If you are not sure, read the appropriate sections before making your selection.

☐ Life sciences ☐ Behavioural & social sciences ☒ Ecological, evolutionary & environmental sciences

For a reference copy of the document with all sections, see nature.com/documents/nr-reporting-summary-flat.pdf

Ecological, evolutionary & environmental sciences study design

All studies must disclose on these points even when the disclosure is negative.

Study description	Using an integrated simulation model, we undertook a comprehensive assessment of China's future sustainable development subject to five major disruptors, including pandemic disease, ageing and shrinking population, deglobalization, climate change, and biodiversity loss. We quantified the potential consequences of China's five major disruptors for sustainable development, and assessed a range of policies for accelerating progress towards the Sustainable Development Goals (SDGs).
Research sample	To provide insights into the robust assessment results of SDG progress, the impact of disruptors, and the effectiveness of policy portfolios in promoting long-term sustainability, we constructed a wide range of scenarios representing both socioeconomic development and policy possibilities of the future. Each scenario was a combination of a state of the world and a policy portfolio.
Sampling strategy	We developed a total of 3,001 states of the world involving no disruptors, only one disruptor, and all disruptors. We then constructed 243 sets of policy portfolios related to education, health, energy, water, and land. By combining 3,001 states of the world with 243 policy portfolios, we obtained 729,243 scenarios.
Data collection	The model was calibrated with historical time series data from 2000 to the most recent year for a selection of variables. Data were sourced primarily from official government sources, such as China Statistical Yearbook and Finance Yearbook of China, as well as official data from international databases hosted by International Monetary Fund, World Bank, and other official sources.
Timing and spatial scale	The study is based on an integrated simulation model calibrated with a wide array of historical time. As for the output, we provided annual simulation results of China's SDG progress from 2022 to 2050.
Data exclusions	We did not perform data exclusions.
Reproducibility	We released the codes to support the reproduction, which are available at the public repository (https://doi.org/10.5281/zenodo.11897708).
Randomization	Randomization is not relevant in our study since we don't need to use this method.
Blinding	Blinding is not relevant in our study since we don't need to use this method.

Did the study involve field work? ☐ Yes ☒ No

Reporting for specific materials, systems and methods

We require information from authors about some types of materials, experimental systems and methods used in many studies. Here, indicate whether each material, system or method listed is relevant to your study. If you are not sure if a list item applies to your research, read the appropriate section before selecting a response.

Materials & experimental systems

n/a	Involved in the study
☒	☐ Antibodies
☒	☐ Eukaryotic cell lines
☒	☐ Palaeontology and archaeology
☒	☐ Animals and other organisms
☒	☐ Clinical data
☒	☐ Dual use research of concern
☒	☐ Plants

Methods

n/a	Involved in the study
☒	☐ ChIP-seq
☒	☐ Flow cytometry
☒	☐ MRI-based neuroimaging

Plants

Seed stocks	N/A
Novel plant genotypes	N/A
Authentication	N/A
